# Neuronal Genes for Subcutaneous Fat Thickness in Human and Pig Are Identified by Local Genomic Sequencing and Combined SNP Association Study

**DOI:** 10.1371/journal.pone.0016356

**Published:** 2011-02-02

**Authors:** Kyung-Tai Lee, Mi-Jeong Byun, Kyung-Soo Kang, Eung-Woo Park, Seung-Hwan Lee, Seoae Cho, HyoYoung Kim, Kyu-Won Kim, TaeHeon Lee, Jong-Eun Park, WonCheoul Park, DongHyun Shin, Hong-Seog Park, Jin-Tae Jeon, Bong-Hwan Choi, Gul-Won Jang, Sang-Haeng Choi, Dae-Won Kim, Dajeong Lim, Hae-Suk Park, Mi-Rim Park, Jurg Ott, Lawrence B. Schook, Tae-Hun Kim, Heebal Kim

**Affiliations:** 1 Division of Animal Genomics and Bioinformatics, National Institute of Animal Science, Rural Development Administration, Suwon, Republic of Korea; 2 Interdisciplinary Program in Bioinformatics, Seoul National University, Seoul, Republic of Korea; 3 Department of Agricultural Biotechnology and Research Institute for Agriculture and Life Sciences, Seoul National University, Seoul, Republic of Korea; 4 Genome Research Center, Korea Research Institute of Bioscience and Biotechnology, Daejeon, Republic of Korea; 5 Division of Applied Life Science, Gyeongsang National University, Jinju, Republic of Korea; 6 Division of Malaria and Parasitic Diseases, National Institute of Health, Seoul, Republic of Korea; 7 Beijing Institute of Genomics, Beijing, China; 8 Institute for Genomic Biology, University of Illinois at Urbana-Champaign, Urbana, Illinois, United States of America; Aarhus University, Denmark

## Abstract

Obesity represents a major global public health problem that increases the risk for cardiovascular or metabolic disease. The pigs represent an exceptional biomedical model related to energy metabolism and obesity in humans. To pinpoint causal genetic factors for a common form of obesity, we conducted local genomic *de novo* sequencing, 18.2 Mb, of a porcine QTL region affecting fatness traits, and carried out SNP association studies for backfat thickness and intramuscular fat content in pigs. In order to relate the association studies in pigs to human obesity, we performed a targeted genome wide association study for subcutaneous fat thickness in a cohort population of 8,842 Korean individuals. These combined association studies in human and pig revealed a significant SNP located in a gene family with sequence similarity 73, member A (FAM73A) associated with subscapular skin-fold thickness in humans (rs4121165, GC-corrected *p*-value  = 0.0000175) and with backfat thickness in pigs (ASGA0029495, *p*-value  = 0.000031). Our combined association studies also suggest that eight neuronal genes are responsible for subcutaneous fat thickness: NEGR1, SLC44A5, PDE4B, LPHN2, ELTD1, ST6GALNAC3, ST6GALNAC5, and TTLL7. These results provide strong support for a major involvement of the CNS in the genetic predisposition to a common form of obesity.

## Introduction

The pig (*Sus scrofa domesticus*) was domesticated from *Sus scrofa*, the wild boar, approximately 9,000 years ago in multiple regions of the world [1], [2]. It has become an important animal as one of the major animal protein sources for humans and is also an exceptionally relevant biomedical model for energy metabolism and obesity in humans since it is devoid of brown fat postnatally and due to its similar metabolic features, cardiovascular systems, and proportional organ sizes [3].

Obesity is increasing in an epidemic manner and represents a major public health problem by increasing risk to cardiovascular disease [4], [5] and metabolic disease such as type 2 diabetes [6]. Recently, two genome-wide association (GWA) studies have expanded the number of genetic susceptibility loci for obesity by identifying SNPs associated with body mass index (BMI) and weight, thus, contributing to obesity risk. The loci identified are located in or near ten genes including the neuronal growth regulator 1 (NEGR1) genes [7], [8]. Both of the GWA studies hypothesized a role of the central nervous system (CNS) in the predisposition to a common form of obesity as has previously been shown for rare monogenic forms of obesity. Although both, BMI and weight are highly heritable, the variants detected in these large GWA studies explained only a small fraction of the inherited variability in BMI and weight [7], [8].

**Figure 1:**
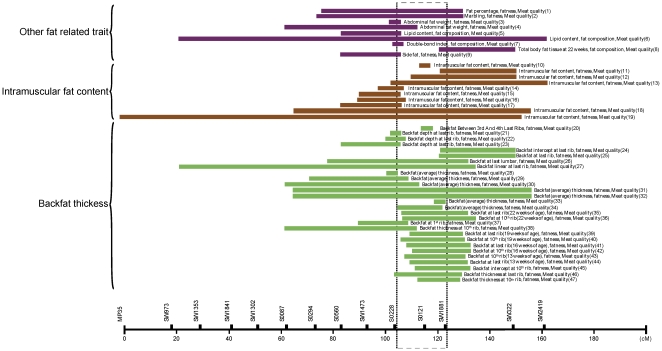
A Pig QTL 18 Mb region affecting fatness and meat quality on SSC6. The information was compiled from PigQTLdb (http://www.genome.iastate.edu/cgi-bin/QTLdb/SS/index). The QTL region and confidence interval of the 18 Mb region is displayed. Reference(s) of each QTL indexed by number in parenthesis are in **Table S1**.

In pigs, high heritability has been estimated for backfat thickness (BFT) and intramuscular fat (IMF) content. Estimates of heritability for BFT are between 50% and 70%, and those for IMF content between 38% and 67% [9]. IMF is necessary to increase meat quality. However, a conflicting relationship exists between IMF and BFT because extra fat in pigs unnecessarily raises the cost of feed [10]. Various efforts have been made to identify the chromosomal regions influencing BFT and IMF by quantitative trait loci (QTL) analysis on pig chromosome 6 (SSC6) BFT [11], [12], [13] and IMF [14], [15], [16], [17], [18], [19], [20]. Recent QTL analyses revealed that the region between *S0228* and *SW1881* might harbor a highly significant QTL affecting fatness and meat quality traits on SSC6 [21], [22], [23] (**Figure 1**). Thus, we carried out both a *de novo* local genomic sequencing and a SNP association study in this region to identify loci associated with BFT and IMF content. Our rationale was that it is important to both identify causal genetic factors in the pig QTL region, and to expand the knowledge of genetic risk factors predisposing to common forms of obesity in humans. To relate and expand the QTL results observed in pigs to human common forms of obesity, we performed a targeted genome wide association study with subcutaneous fat thickness in a cohort population of 8,842 Korean individuals.

## Results

### Local genomic sequencing of the pig QTL region

**Figure 2:**
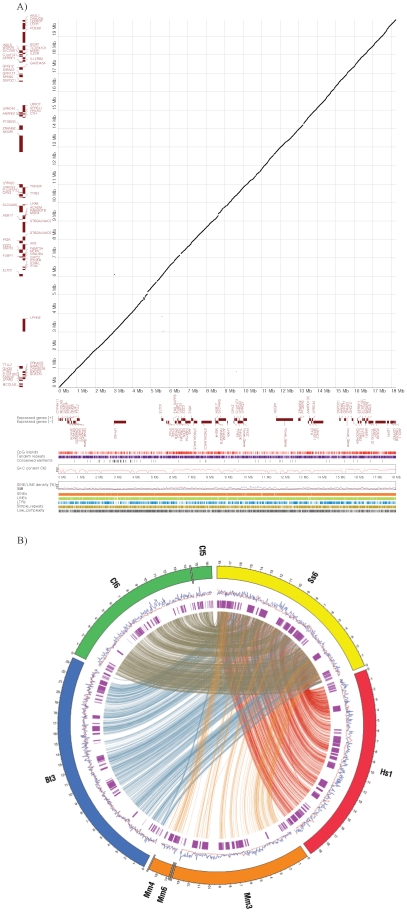
Comparative genomic analysis of pig QTL region affecting fatness and meat quality on SSC6. Similar genomic structure and contents was revealed by comparative analysis between the human syntenic region and the pig QTL region (A) and visualization of syntenic blocks along the region between pig (Ss) and the four other species: human(Hs), mouse(Mm), dog(Cf) and cow(Bt) (B). In the dot-plot analysis (A), protein coding genes are shown as forward (upper) and reverse (lower) in brown color according to x- and y-axis for pigs and human, respectively. The conserved segments from 70-100% are plotted. The genomic feature of pig appear in order from top to bottom under dot-plot: CpG islands, Tandem repeats, G+C content, SINE (blue) and LINE (red) repeat densities using a sliding window of 100 kbp, Interspersed repeat elements (SINEs, LINEs, LTR elements, Simple repeats and Low complextiy). In the synteny maps (B), the rings depict from outside to inside: synteny regions of each chromosome, SINE (blue) and LINE (red) repeat density using a sliding window of 100 kb and protein-coding genes (purple). Genomic coordinates are shown in 100 kb intervals. Synteny blocks larger than 5 kb are displayed by connecting lines.

To perform *de novo* local genomic sequencing of the QTL region, a total of 316 markers were developed from pig Bacterial Artificial Chromosome (BAC) end sequences summarized in **Table S2**. The Korean Native Pig (KNP) BAC library [24] was screened by 4D-PCR [25], and sequenced by a shotgun strategy. Additional BAC clones produced by the International Swine Genome Sequencing Consortium were used for gap filling. Approximately 18.2 Mb sequence was generated after assembling these sequences. Out of 72 protein coding genes on human syntenic regions, 70 orthologous genes were detected in pigs. Three porcine pseudo-genes and four novel genes were annotated in this region. A total of 10 non-coding RNAs were detected (**Table S3**). Comparative analysis with the human syntenic region exhibited a genomic structure and contents similar to that in the pig QTL region (**Figure 2A**), which included functional genes, repetitive elements, GC content and CpG islands. A detailed description of the genomic sequencing is provided in the methods. The similarity of the genomic structure and contents with the human syntenic region augments the merit of the pig as a relevant biomedical model to identify and expand causal genetic factors predisposing to common forms of human obesity. Visualization of syntenic blocks along the region between pig and four other species, human, mouse, dog and cow, shows closer genomic similarity between pig and human than between pig and mouse (**Figure 2B**). Rodent models have long been the pillar of obesity and metabolic syndrome research. However, marked differences in metabolism and adipose tissue biology between rodents and humans have been recognized and, thus, the pig is emerging as a more appropriate biomedical model for obesity in humans because of its biological similarities with humans [3].

### SNP association studies for backfat thickness and intramuscular fat content in pigs

**Figure 3:**
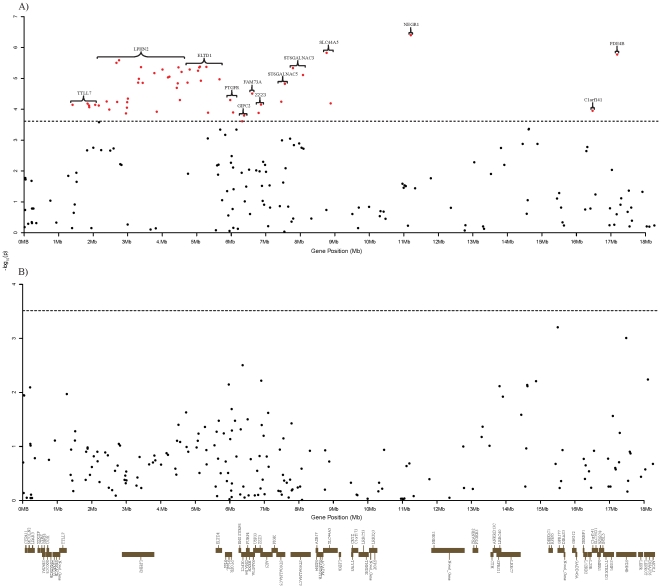
–log_10_(*p*-value) of SNPs in the 18.2 Mb pig genomic region. 52 SNPs were identified to be associated with backfat thickness trait (A), and none of the SNPs were found to be significantly associated with intramuscular fat content (B). Gene locations are shown by bars and gene symbols beneath the figure. Genes on the same side indicate same transcriptional direction of the genes.

After filtering SNP genotypes for quality control (Materials and Methods), we conducted a SNP association study of the region using 235 SNPs in 527 pigs for BFT and IMF content traits. We identified 52 SNPs (22.1% of the 235 SNPs) associated with BFT at *p* = 0.000213, equivalent to *p-*value  = 0.05 after Bonferroni correction (**Figure 3A** and **Table S4**). Although this genomic region has been suggested to contain QTLs for BFT, more significant SNPs than expected were identified considering that the Bonferroni procedure is a very conservative correction (some of the SNPs are in strong linkage disequilibrium and therefore the tests are not independent). None of the SNPs, however, were found to be significantly associated with IMF content (**Figure 3B**). The significant SNPs are located in or near 13 protein coding genes, with 10 genes containing at least one significant SNP each. The SNP (ALGA0122230) showing the strongest association with BFT is located near the neuronal growth regulator 1 (NEGR1) gene. Recently the GIANT consortium [8] reported that the NEGR1 obesity-associated SNPs they detected seemed to be in strong linkage disequilibrium with nearby copy number variations (CNV), although there is currently no functional evidence to support the involvement of the CNV in non-syndromic human obesity. The NEGR1 protein participates in the regulation of neurite outgrowth in the developing brain [26], [27]. Interestingly, we found that of the 13 genes associated with BFT, 8 genes including NEGR1 are involved in psychiatric disease, neural development or high expression in CNS. The eight genes include: NEGR1, a member of solute carrier (SLC) superfamily 44 (SLC44A5); phosphodiesterase 4B (PDE4B); latrophilin 2 (LPHN2); epidermal growth factor; latrophilin; seven transmembrane domains containing 1 (ELTD1), ST6 (α-*N*-acetylneuraminyl-2,-3-β-galactosyl-1,3)-*N*-acetylgalactosamine-α-2,6-sialyltransferase 3 (ST6GALNAC3), ST6GALNAC5; and tubulin tyrosine ligase-like family, member 7 (TTLL7).

The SLC superfamily is a major group of membrane transporter proteins that control cellular uptake and efflux of nutrients, neurotransmitters, metabolites, drugs, and toxins [28]. Although biological and neurological functions for the majority of the SLC genes in the mammalian brain are largely unknown, recently, Dahlin *et al.* [29] reported that 82% of known SLC genes were expressed in the brain. Among the members of this superfamily, a member of SLC44 was present in oligodendrocytes. To date, the biological function of SLC44A5 is unknown. PDE4B belongs to a family of four PDE4 genes, all coding for phosphodiesterases that hydrolyze the second messenger cyclic adenosine monophosphate (cAMP). Since PDE4B was first suggested as a risk factor for schizophrenia [30], PDE4B has also been suggested as a candidate gene associated with both schizophrenia and bipolar disorder [31]. Variation in the resting electroencephalogram (EEG) is associated with common, complex psychiatric traits including alcoholism, schizophrenia, and anxiety disorders [32]. Recently, genome-wide association identified SNPs with significant association to EEG traits on 1p31.3 of human chromosome 1 [32], and interestingly, it is the human syntenic region to the pig QTL region in which ST6GALNAC3 and LPHN2 are included. LPHN2 is a G-protein-coupled receptor related to the receptor that binds black widow (*Latrodectus*) spider venom in synaptic membranes [33]. ST6GALNAC3 is an integral Golgi membrane protein, which catalyzes the transfer of sialic acids to carbohydrate groups on glycoproteins and glycolipids. Expression of the ST6GALNAC5 gene is normally restricted to the brain both in mice [34] and humans [35]. Based on a linkage study [36] the ELTD1 gene has been implicated in neuropeptide signaling and signal transduction pathways, making it an important candidate for genetic risk to cannabis use disorders. TTLL7 is a highly specific enzyme that performs β-tubulin polyglutamylation [37] and has been suggested as a candidate gene associated with Alzheimer's disease [38].

### Targeted genome wide association study with subcutaneous fat thickness in a cohort population

**Figure 4:**
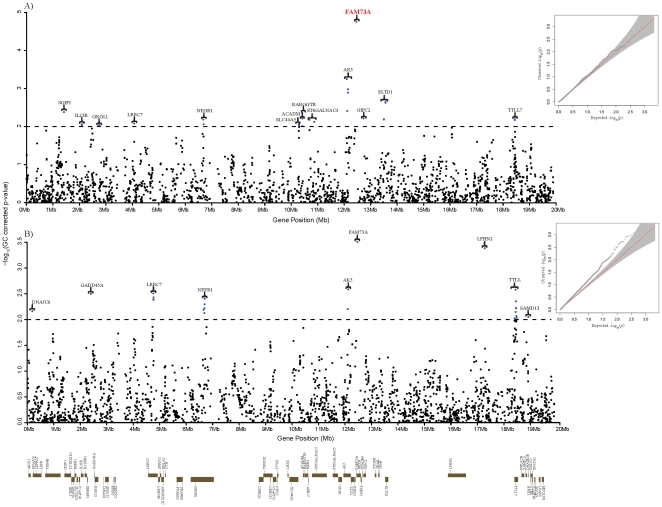
–log_10_(Genomic control-corrected *p*-value) of 2,143 SNPs associated with SUB (A) and SUP (B) in the human syntenic region. Using a false discovery rate (FDR) *q* value <0.05 [41], a SNP located in FAM73A gene is significantly associated with SUB indicated in red. The genomic control parameter λ value in SUB-SNP association study was 1.037 and the λ value in SUP-SNP association study was 1.187 indicated by the QQ-plots. Genomic control-corrected *p*-value threshold of 0.01 is indicated by the dotted line.

In order to relate these 13 functional genes including 8 neuronal genes to non-syndromic human obesity, we carried out a SNP association study for subcutaneous fat content in the human syntenic region using the recently reported 8,842 individuals of a Korean cohort data [39]. Unlike BFT measurement in pigs, the subcutaneous fat in humans was indirectly measured by subscapular and suprailiac skin-fold thickness (SUB and SUP). A total of 2,143 SNPs passed all quality control filters in the human syntenic region (Materials and Methods). The genomic control parameter λ value in SUB-SNP association study was 1.037, indicating no overall inflation of statistical results due to population stratification, while the λ value in the SUP-SNP association study was 1.187 (**Figure 4**). After genomic control (GC) correction [40], no evidence of inflation remained for either of the association studies. Using a false discovery rate (FDR) *q* value [41] <0.05, we identified one SNP located in a gene family with sequence similarity 73, member A (FAM73A) gene associated with SUB (rs4121165, GC-corrected *p*-value  = 0.0000175) (**Figure 4A** and **Table S5**). The FAM73A gene was also significantly associated with BFT in pigs. Considering that SUB is measured in the human back, FAM73A is a strong candidate gene responsible for subcutaneous back fat thickness in both humans and pigs. The SNP also showed the strongest association with SUP. To our knowledge, no biological function of the gene has been reported. However, based on tissue expression analysis in humans of the GeneCards (www.genecards.org), the gene seems to be expressed prominently in the nervous system. After the FDR correction, there were no significant SNPs associated with the two skin fold thickness measurements except the SNP in the FAM73A gene. However, using a GC-corrected *p*-value threshold of 0.01, genes containing SNPs with a lower *p*-value cutoff seem to show enrichment of the 13 genes (**Table S4**) associated with pig BFT. Using the threshold, out of 14 genes containing significant SNPs in the SUB-SNP association, 7 genes are among 13 functional genes associated with pig BFT. Likewise in the SUP-SNP association, 4 genes out of 9 belong to those 13 functional genes (**Figure 4B** and **Table S6**). Considering 72 protein coding genes in the region (probability of success  = 13/72), exact binomial probability observing the given number of genes or more in each result, 7 out of 14 genes, is 0.0065 for SUB-SNP association and 0.0621 for SUP-SNP association which is 4 out of 9 genes. Therefore, it is unlikely to observe this number of common genes by chance in both, the human and pig association studies especially for the SUB-SNP association.

## Discussion

Measurement error in assessing the skin-fold thickness may be considerably larger than the BFT measurement in pigs. Skin-fold thicknesses in human are affected by individual and regional differences in compressibility that vary with age, gender and recent weight loss. In addition, pressure from skin-fold caliper measurement may force some adipose tissue lobules to slide into areas of lesser pressure. This sliding may be more marked for thick skin-folds in which the adipose tissue contains little connective tissue [42]. On the contrary, BFT in pigs are accurately measured with a ruler between the 10^th^ and 11^th^ rib on the chilled carcass. Because of these measurement errors, the association study in pigs is likely to provide higher statistical power than that for humans if they are under similar conditions except the measurement errors.

**Figure 5:**
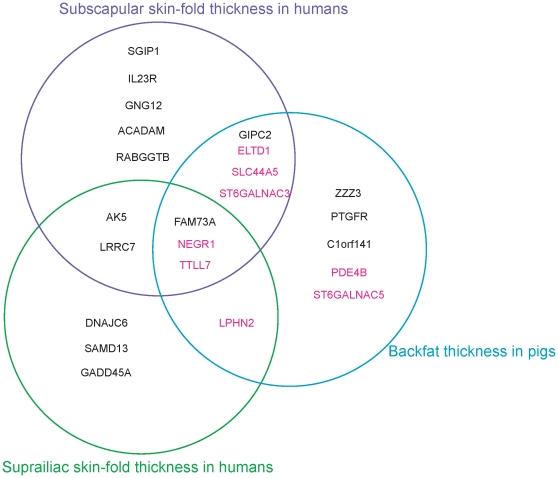
Summary of genes identified in our combined association studies in humans and pigs in the predefined fatness related pig QTL region. Intersection of the three association studies show three most likely genes, FAM73A, NEGR1 and TTLL7, as being responsible for genetic predisposition to common forms of obesity, especially subcutaneous fat thickness. Eight neuronal genes identified in the pig SNP-BFT association study are indicated in pink.

Our combined association studies in human and pig in the predefined fatness related pig QTL region revealed three most likely genes, FAM73A, NEGR1 and TTLL7, as being responsible for genetic predisposition to common forms of obesity, especially subcutaneous fat thickness (**Figure 5**). The second likely set of genes for genetic predisposition includes five genes, LPHN2, SLC44A5, ELTD1, ST6GALNAC3 and GIPC2 (**Figure 5**). As mentioned above, two recent GWA studies [7], [8] suggested the role of the CNS in the predisposition to the non-syndromic form of obesity. Our results strongly support a major involvement of the CNS in the genetic predisposition, and suggest several neuronal genes as genetic risk factors for the polygenic common form of obesity (**Figure 5**). Except for the NEGR1 gene, to our knowledge, the other neuronal genes are newly suggested in our research for the genetic association with obesity related traits. Our findings of candidate causal genes may provide expanded insight into mechanisms underlying obesity biology. Further evaluation of these candidate genes in humans and pig may enable researchers to accelerate gaining knowledge of genetic factors for common forms of obesity.

## Materials and Methods

### Ethics statement

Approval was granted from relevant review boards in all study sites; all included subjects gave informed written consent. The Korea Centers for Disease Control and Prevention's review board reviewed and approved the Korean SNP association studies. For the pigs experiment, the study protocol and standard operating procedures were reviewed and approved by the National Institute of Animal Science's Institutional Animal Care and Use Committee (No. 2009-077, C-grade).

### Pig BAC sequencing and assembling

BAC clones consisting of the 18.2 Mb contigs were screened from the Korean Native Pig BAC library [43]. The pig BAC end sequences (BES) corresponding to the syntenic region between 65 Mb and 85 Mb of human chromosome 1 were obtained from Sus scrofa Project site of the Wellcome Trust Sanger Institute (http://www.sanger.ac.uk/cgi-bin/Projects/S_scrofa/BESsearch.cgi). A total of 316 markers were developed from pig BESs with intervals of 60 kb to screen BAC clones (**Table S2**). The BAC library was pooled for 4D-PCR screening [25]. The 4D-PCR screening consisted of a two-step screening process: the first PCR was performed on master pools and the second on plate row/column, well row/column pools in a total volume of 15 µL with 10 ng in each pool. PCR amplifications were performed in a PTC 200 thermocycler (MJ Research, USA). Thermal cycling parameters were defined as follows: predenaturation at 95°C for 2 min; followed by 32 cycles of 95°C for 30 s, annealing temperature for 30 s, and 72°C for 30 s; and then a final step at 72°C for 5 min. PCR products were separated on a 2% agarose gel containing ethidium bromide and visualized using a UV light source.

The screened BAC clones were sequenced by a shotgun strategy. The BAC DNAs were isolated using the Large Construct Kit (Qiagen, USA). A total of 15 µg BAC DNA was used to obtain random fragments of 2∼3 kb. Fragmentation was performed using the HydroShear DNA Shearing Device (Genomic Solution, USA) with the following parameters: 200 µL volume of DNA solution, 11 speed code, and 20 cycles. Small sizes of the fragments were removed using the Sizesep 400 spin column (Amersham Biosciences, USA) and CHROMA SPIN+TE1000 (Clontech, USA) and were subsequently repaired with DNA polymerase and the polynucleotide kinase method (BKL Kit; TaKaRa, Japan). The prepared DNA fragments were cloned into the dephosphorylated SmaI site of pUC19 (Qbiogene, USA). Ligates were transformed into DH10B by electroporation (Gene Pulser II, Bio-Rad, USA). Approximately 1000 plasmids in each shotgun DNA library were randomly selected for sequencing. Plasmid DNAs were bi-directionally sequenced for each plasmid with the BigDye Terminator v3.1 Cycle Sequencing Kit (Applied Biosystems) and the ABI 3730 automatic sequencer (Applied Biosystems). Sequence data were assembled using the PHRAP program (University of Washington, Seattle, WA, USA). To fill gaps, primer pairs were designed from the high-quality region with a PHRAP score greater than 70 on both ends of each contig. PCR amplifications were performed using appropriate BAC DNA used to construct the shotgun library as a template. PCR products were inserted into the pGEM T Easy vector (Promega, USA) and sequenced. Finishing assembly was performed in Seqman (DNASTAR, USA). The complete sequences of 126 KNP BAC clones and 33 unfinished KNP BAC clones were deposited into EMBL/GenBank (EF488234, FN673706-FN673830, FN674549-FN675238). A total of 29 CHORI242 BAC clones were selected from the FPC clone map of the Wellcome Trust Sanger Institute website to fill the gaps within the KNP BAC clone maps (**Table S1**). The selected CHORI242 BAC clones were sequenced to up to 8-fold depth (FN677038-FN677340).

All BAC clone sequences were assembled with Seqman to construct continuous genomic sequences. The representative genome sequence of approximately 18.2 Mb mainly consisted of KNP BAC clone sequences. The pig genome sequences of Sscrofa9 produced by Swine Genome Sequencing Consortium were used to replace the remaining gaps within the BAC clone contigs (ftp://ftp.ensembl.org/pub/current_fasta/sus_scrofa/dna/).

### Sequence annotation and comparative genome analysis

The genomic sequence of 18,261,618 bp was used to predict putative genes using de novo gene prediction programs, that is, GENSCAN [44], AUGUSTUS [45] and GeneMark.hmm [46]. For cis-alignment analysis, the *Sus scrofa* UniGene build 38 and expressed sequence tag (EST) sequences were downloaded from NCBI. Then, the ESTs were aligned against the sequenced genome using BLAT [47] and filtered by 98% coverage cutoff. For trans-alignment, human and mouse protein sequences from the UniProtKB database (http://www.uniprot.org/downloads) were aligned against the sequenced genome using BLAT [47] and filtered by 95% coverage cutoff. To eliminate false positive gene findings, *de novo* genes which included EST-aligned or trans-aligned genomic region were selected in the annotation process. Final gene annotation was determined by careful manual inspection.

To perform comparative genome analysis, we downloaded the assembled genome sequences: Human (1∶65,592,395-85,484,668); Mouse (4∶101,091,882-102,963,468, 6∶67,372,926-66,971,344, 3∶145,804,770-159,502,701); Cow (3∶62,655,270-86,200,728); Dog (5∶46,172,737-48,121,421, 6∶65,987,112-80,443,206) from the UCSC Genome Browser [48]. The numbers in each parenthesis indicate chromosome number and bp locations of the chromosome. To mask interspersed repeats and low complexity regions, the RepeatMasker program (http://www.repeatmasker.org) was used with the -xsmall option for each corresponding library. With masked genome sequences, the BLASTZ alignment program [49] was used to define the map of conserved synteny using the “C = 2 T = 1 H = 2200 Z = 10” option to align each of the genomes to the Pig sequence. GC content density was calculated by using 100 kb non-overlapping bins along each chromosome. For identifying clusters of CpG dinucleotides in GC content-rich regions, we used the CpG island searcher program (CpGi130) with criteria (GC content >50%, ObsCpG/ExpCpG >0.60, and length >200 bp) [50]. To visualize the global distribution of synteny blocks along the genome, we used the CIRCOS visualization program [51] and a dotplot analysis program, custom-made with a perl script using BLASTZ result.

### Pigs and SNP association studies with backfat thickness and intramuscular fat traits

Five Korean native (domesticated wild) sires and ten Landrace dams were used to produce a three-generation pedigree at the National Institute of Animal Science, Rural Development Administration (RDA). Among the F1s, ten boars were randomly chosen and mated with up to six F1 sows to generate 38 full-sib F2 families [52], [53]. DNA samples were obtained from a total of 527 F2 progeny and genotyped with the iSelect Infinium Porcine ArrayChips (Illumina, San Diego, CA, USA). Intramuscular fat content (IMF) and backfat thickness (BFT) traits were analyzed to find the significant SNPs on an 18.2 Mb genomic sequence between SW2098 and SW1881 on pig chromosome 6. IMF was determined in a sample of longissimus muscle, and BFT was measured between the 10^th^ and 11^th^ rib. Average values (± standard deviation) for the IMF and BFT in the F2s were 2.21% (±2.76%) and, 24.1 mm (±8.1 mm) respectively [52], [53]. Genomic DNA was isolated from blood samples using the Wizard Genomic DNA Purification Kit (Promega, Madison, WI, USA). A total of 451 SNPs out of 62,163 SNP probes were mapped on the 18.2 Mb genomic sequence (88,750,779 bp-103,764,358 bp) by BLAT (minIdentity = 97, tGapCount ≤1 and 3′end match).

A goodness of fit chi-square test was used to test Hardy-Weinberg equilibrium (HWE) by comparing the observed number of subjects for each genotype with the expected number of subjects assuming HWE and so genotype distributions were tested at each polymorphic locus for departure from HWE. SNPs were screened out at *p*-value <0.001 via HWE tests. We excluded SNPs with >5% missing genotypes and with minor allele frequencies <5%. As a result, 235 SNPs in chromosome 6 passed our quality control filters and a total of 527 pig individuals were included in the analysis.

SNP association analyses of the 18.2 Mb region with BFT and IMF in the pigs were performed using the genomewide rapid association mixed model and regression (GRAMMAR) approach [54]. The basic idea of this GRAMMAR is to perform a single polygenic analysis using the complete pedigree but ignoring marker data. Subsequently, residuals from the polygenic analysis, which are adjusted for polygenic covariation and fixed effects are used as the quantitative phenotype for whole genome association study. In the initial step, the data are analyzed under the mixed model in ASREML [55]. 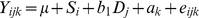
(1)where *Y_ijk_* is the trait measured in the *k^th^* animal of *i^th^* sex and *j^th^* age at slaughter days; µ is an overall mean, *S_i_* is the fixed effect of *i^th^* sex, *b_1_* is a regression coefficient, *D_j_* is covariate for the age at slaughter days, *a_k_* is additive genetic (polygenic) effect of *k^th^* animal, fitted as a random effect and *e_ijk_* is the random residual error. The variance for additive genetic (polygenic) effects of animals is defined as *Var* (a)  =  *A*σ_a_ based on the pedigree of the offspring and σ_a_ is the additive genetic variance due to polygenes [56]. For the residual random effects, the variance is defined as *I*σ^2^_e,_ where *I* is the identity matrix and σ^2^_e_ is the residual variance. The residuals from this analysis are given by 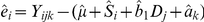
where 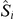 and 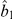 are the estimates of sex and age effects and 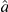 *_j_* is the estimated contribution from the polygene (breeding value). In the second step for the whole genome association study, these residuals are used as the phenotype in a simple linear regression for each SNP (g *_i_*),
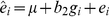
(2)where 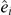 is the vector of residuals from model (1), µ is the mean, *g* is the vector of genotypes at the marker *i*, *b_2_* is the marker genotype effect and 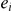 is the vector of random residuals. This approach is called GRAMMAR [54].

The simple linear regression analysis was performed in the R/SNPassoc package [57]. Markers with a test statistic exceeding a threshold corresponding to a p-value below the region-wise Bonferroni corrected significance threshold (0.05/#SNPs) were selected for the final test using the full animal model in ASREML [55]:
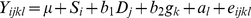
(3)where *Y_ijkl_* is the trait measured in the *l^th^* animal of *i^th^* sex, *j^th^* age at slaughter days and *k^th^* genotype; µ is an overall mean, *S_i_* is the fixed effect of *i^th^* sex, *b_1_* is a regression coefficient, *D_j_* is covariate for the age at slaughter days, *b_2_* is the marker genotype effect, *g_k_* is the vector of genotypes at the marker *k*, *a_l_* is additive genetic (polygenic) effect of *l^th^* animal, fitted as a random effect and *e_ijkl_* is the random residual error. Additive genetic covariances among animals and residual variance are described in the model (1).

### SNP association study of subcutaneous fat thickness in human

The Korea Association Resource (KARE) project was initiated in 2007 to undertake large-scale GWA analyses. Participants in this project were recruited from two community-based cohorts (i.e., the rural Ansung and urban Ansan cohorts) in the Gyeonggi Province of South Korea. The Ansung and Ansan cohorts consist of 5,018 and 5,020 participants, respectively, ranging in age from 40 to 69 years. Genomic DNAs was isolated from peripheral blood drawn from the participants and genotyped on the Affymetrix Genome-Wide Human SNP array 5.0 containing 500,568 SNPs. Prior to the analysis, we performed genotype calling and quality control as previously described in Cho *et al*. [39]. After sample and SNP quality controls, a total of 8,842 individuals and 2,143 SNPs in the human syntenic region were included in the association studies. Subcutaneous fat in humans was indirectly measured by subscapular and suprailiac skin-fold thickness (SUB and SUP) for the SNP association study. To perform a SNP association study with skin-fold thickness measurement, data transformation of the actual skin-fold thickness measurement is desirable because the frequency distribution of most skin-fold measurements is skewed, and the relationship of body density to skin-folds may not be rectilinear because of a larger proportion of the body fat which is deposited subcutaneously with increasing obesity [58]. Natural logarithmic transformation for SUB measurement and square root transformation for SUP measurement was performed in which the assumption of normal distribution was more reasonable for each trait. Linear regression analysis was performed in an additive model using PLINK [59], including sex, age and geographic region as covariates. The *p*-values were adjusted by a genomic control method [40] and followed by FDR [41] corrections as implemented in PLINK [59]. As described in the result, we could not find significant SNP associations except one SNP of the traits. Thus we used GC-corrected *p*-value threshold of 0.01 to summarize the top highest SNP associations with the traits in human and test enrichment of significant genes in pigs.

## Supporting Information

Table S1 References of each QTL region indicated in the Figure 1.
(DOC)

Table S2 List of sequence-tagged sites (STSs) designed used to screen bacterial artificial chromosome (BAC) clones. The STSs were designed from BAC end sequences (BES) mapped on PigMap corresponding to human genomic region between 65 Mb and 85 Mb in chromosome 1.
(DOC)

Table S3 List of gene annotation in the pig 18.2 Mb region.
(DOC)

Table S4 List of SNPs significantly associated with the backfat thickness trait in the 18.2 Mb region.
(DOC)

Table S5 List of SNPs associated with subscapular skin-fold thickness at the threshold of genomic control-corrected *p*-value 0.01.
(DOC)

Table S6 List of SNPs associated with suprailiac skin-fold thickness at the threshold of genomic control-corrected *p*-value 0.01.
(DOC)
