# Supplementary material for: Neuronal Genes for Subcutaneous Fat Thickness in Human and Pig Are Identified by Local Genomic Sequencing and Combined SNP Association Study
Source: PLoS One. 2011 Feb 2;6(2):e16356. doi: 10.1371/journal.pone.0016356 (PMC3032728; doi:10.1371/journal.pone.0016356)
Supplement: Table S1 — References of each QTL region indicated in the Figure 1. (DOC) [file pone.0016356.s001.doc]

**Table S1**. References of each QTL region indicated in the Figure 1.

| Number | Traits | QTL span | References |
| --- | --- | --- | --- |
| 1 | Fat percentage | 76.1-129.2 (cM) | D.B. Edwards et al., J. Anim. Sci. (2008) 86:254-266 |
| 2 | Marbling | 74.3-129.2 (cM) | D.B. Edwards et al., J. Anim. Sci. (2008) 86:254-266 |
| 3 | Abdominal fat weight | 62.8-112 (cM) | G.Yue et al., J Anim Breed Genet. (2003) 120(1); 45-55 |
| 4 | Abdominal fat weight | 102.2-105.1 (cM) | G.Yue et al., J Anim Breed Genet. (2003) 120(1); 45-55 |
| 5 | Lipid content | 83.3-105.2 (cM) | M. Mohrmann et al., Animal Genetics. (2006) 37: 435-443 |
| 6 | Lipid content | 21.9-161.3 (cM) | D.B. Edwards et al., J. Anim. Sci. (2008) 86:241-253 |
| 7 | Double-bond index | 105 (cM) | Clop A et al., Mamm Genome.(2003) Sep;14(9):650-6 |
| 8 | Total body fat tissue (22 weeks of age) | 149.8-121.1 (cM) | D.B. Edwards et al., J. Anim. Sci.(2008) 86:241-253 |
| 9 | Side fat | 83.3-105.2 (cM) | M. Mohrmann et al., Animal Genetics. (2006) 37: 435-443 |
| 10 | Intramuscular fat content | 114.8 (cM) | Ovilo C et al., Mammalian genome. (2000) 11 (4): 344-6 |
| 11 | Intramuscular fat content | 121.1-149.8 (cM) | M. Mohrmann et al., Animal Genetics. (2006) 37: 435-443 |
| 12 | Intramuscular fat content | 110.4-149.8 (cM) | Paszek, A A et al., Animal biotechnology. (2001) 12 (2): 155-65 |
| 13 | Intramuscular fat content | 102-161.4 (cM) | de Koning DJ et al., Genetics. (1999) Aug;152(4):1679-90 |
| 14 | Intramuscular fat content | 97-106.1 (cM) | Ovilo Cet al., J Anim Sci. (2002) Nov;80(11):2801-8 |
| 15 | Intramuscular fat content | 90.7-105.2 (cM) | Szyda J et al., J Appl Genet. (2002) 43(1):69-83 |
| 16 | Intramuscular fat content | 89.3-107 (cM) | de Koning DJ et al., Proc Natl Acad Sci U S A. (2000) Jul 5;97(14):7947-50 |
| 17 | Intramuscular fat content | 83.3-105.2 (cM) | Grindflek E et al., Mamm Genome. (2001) Apr;12(4):299-304 |
| 18 | Intramuscular fat content | 65-155.2 (cM) | Ovilo C et al., Genet Sel Evol. (2002) Jul-Aug;34(4):465-79 |
| 19 | Intramuscular fat content | 0-153 (cM) | Gerbens F et al., J Anim Sci. (2001) Feb;79(2):347-54 |
| 20 | Backfat between 3rd and 4th last ribs | 117.2 (cM) | Kim, J H et al., BMB reports. (2008) 41 (6): 466-71 |
| 21 | Backfat depth at last rib | 102.2-105.1 (cM) | G.Yue et al., J Anim Breed Genet. (2003) 120(1); 45-55. |
| 22 | Backfat depth at last rib | 100-107 (cM) | Varona L et al., Genet Res. (2002) Oct;80(2):145-54 |
| 23 | Backfat depth at last rib | 83.3-105.2 (cM) | M. Mohrmann et al., Animal Genetics. (2006) 37: 435-443 |
| 24 | Backfat intercept at last rib | 149.8-121.1 (cM) | D.B. Edwards et al., J. Anim. Sci. (2008) 86:241-253 |
| 25 | Backfat at last rib | 149.8-121.1 (cM) | D.B. Edwards et al., J. Anim. Sci. (2008) 86:254-266 |
| 26 | Backfat at last lumbar | 78.8-131.2 (cM) | D.B. Edwards et al., J. Anim. Sci. (2008) 86:254-266 |
| 27 | Backfat linear at last rib | 21.9-134.2 (cM) | D.B. Edwards et al., J. Anim. Sci. (2008) 86:241-253 |
| 28 | Backfat weight | 62.8-112 (cM) | G.Yue et al., J Anim Breed Genet. (2003) 120(1); 45-55 |
| 29 | Average backfat thickness | 71-108 (cM) | Varona L et al., Genet Res. (2002) Oct;80(2):145-54 |
| 30 | Average backfat thickness | 62.8-112 (cM) | G.Yue et al., J Anim Breed Genet. (2003) 120(1); 45-55 |
| 31 | Average backfat thickness | 65-155.2 (cM) | Ovilo C et al., Genet Sel Evol. (2002) Jul-Aug;34(4):465-79 |
| 32 | Average backfat thickness | 65-155.2 (cM) | Ovilo C et al., Genet Sel Evol. (2002) Jul-Aug;34(4):465-79 |
| 33 | Backfat (average) thickness - ultra sound | 120.5 (cM) | **G. Muñoz** et al., J. Anim. Sci.(2009) 87 (2): 459-68 |
| 34 | Backfat (average) thickness - by Fat-O-Meater | 105.2-121.1 (cM) | M. Mohrmann et al., Animal Genetics. (2006) 37: 435-443 |
| 35 | Backfat at last rib  (22 weeks) | 107.1-131.2 (cM) | D.B. Edwards et al., J. Anim. Sci. (2008) 86:241-253 |
| 36 | Backfat at tenth rib (22 weeks) | 107.1-134.2 (cM) | D.B. Edwards et al., J. Anim. Sci. (2008) 86:241-253 |
| 37 | Backfat at first rib | 90-108 (cM) | Varona L et al., Genet Res. (2002) Oct;80(2):145-54 |
| 38 | Backfat at tenth rib | 62.8-112 (cM) | G.Yue et al., J Anim Breed Genet. (2003) 120(1); 45-55 |
| 39 | Backfat at last rib  (19 weeks) | 110.7-129.2 (cM) | D.B. Edwards et al., J. Anim. Sci. (2008) 86:241-253 |
| 40 | Backfat at tenth rib (19 weeks) | 110.7-129.2 (cM) | D.B. Edwards et al., J. Anim. Sci. (2008) 86:241-253 |
| 41 | Backfat at last rib  (16 weeks) | 109.5-132.2 (cM) | D.B. Edwards et al., J. Anim. Sci. (2008) 86:241-253 |
| 42 | Backfat at tenth rib (16 weeks) | 111.9-132.2 (cM) | D.B. Edwards et al., J. Anim. Sci. (2008) 86:241-253 |
| 43 | Backfat at tenth rib (13 weeks) | 110.7-131.2 (cM) | D.B. Edwards et al., J. Anim. Sci. (2008) 86:241-253 |
| 44 | Backfat at last rib  (13 week) | 108.3-130.2 (cM) | D.B. Edwards et al., J. Anim. Sci. (2008) 86:241-253 |
| 45 | Backfat intercept at tenth rib | 112.4-132.2 (cM) | D.B. Edwards et al., J. Anim. Sci. (2008) 86:241-253 |
| 46 | Backfat at last rib  (10 weeks) | 104.7-129.2 (cM) | D.B. Edwards et al., J. Anim. Sci. (2008) 86:241-253 |
| 47 | Backfat at tenth rib (10 weeks) | 113.0-128.2 (cM) | D.B. Edwards et al., J. Anim. Sci. (2008) 86:241-253 |
