# Supplementary material for: Neuronal Genes for Subcutaneous Fat Thickness in Human and Pig Are Identified by Local Genomic Sequencing and Combined SNP Association Study
Source: PLoS One. 2011 Feb 2;6(2):e16356. doi: 10.1371/journal.pone.0016356 (PMC3032728; doi:10.1371/journal.pone.0016356)
Supplement: Table S2 — List of sequence-tagged sites (STSs) designed used to screen bacterial artificial chromosome (BAC) clones. The STSs were designed from BAC end sequences (BES) mapped on PigMap corresponding to human genomic region between 65 Mb and 85 Mb in chromosome 1. (DOC) [file pone.0016356.s002.doc]

**Table S2.** List of sequence-tagged sites (STSs) designed used to screen bacterial artificial chromosome (BAC) clones. The STSs were designed from BAC end sequences (BES) mapped on PigMap corresponding to human genomic region between 65 Mb and 85 Mb in chromosome 1.

| **No.** | **Human Build 36** | **BES Source** | **Forward Primer** | **Reverse Primer** | **Size (bp)** | **Korean Native Pig BAC clones** | **CHORI242 clones** |
| --- | --- | --- | --- | --- | --- | --- | --- |
| **1** | **65380317** | **bT120C18SP6** | **CTTGGGCTGATATCTTGTGT** | **AGGTACAGAGTCCAGCAGTG** | **204** | **KNP_185H1(F)^*^** |  |
| **2** | **65435391** | **bE82B9T7** | **GCTCAGAACGAATCTAATGG** | **CTCCCAGATACCGTGAAGTA** | **192** | **KNP_185H1(F)** |  |
| **3** | **65494965** | **bT124C6SP6** | **CGGCGTTTTTACTTTTAGTC** | **CATTCACACAACATCTGGAA** | **201** | **KNP_185H1(F), KNP_466E2(F)** |  |
| **4** | **65555586** | **bT212M6T7** | **ATGCAAGCTTTTTGACTGAG** | **CTGGATCTAGACTAGCCTGTG** | **153** | **KNP_466E2(F)** |  |
| **5** | **65617975** | **bT231P21T7** | **GGCATCTATGCCTTGTAAGT** | **GGGAATGGTCATACTCTGAA** | **205** | **KNP_466E2(F)** |  |
| **6** | **65667588** | **bT195I22T7** | **GCTTGGAGAAAGTGAAACTG** | **ACCACCTTCTCTTCATTGAG** | **199** | **KNP_1159H5(F)** |  |
| **7** | **65712290** | **bT212M6SP6** | **GGAACAAACGTAGATTGCAT** | **GCCAATATACTCCACCTTGA** | **196** | **KNP_1159H5(F), KNP_1686B1(F)** |  |
| **8** | **65761434** | **bT231P21SP6** | **TTTGAACAGCTAAAAATAAATATCA** | **AAATCCAATGGTAGCTTCTG** | **156** | **KNP_1686B1(F)** |  |
| **9** | **65811671** | **bE192I10SP6** | **TAGACAGAGAGCCAAATGGT** | **CTGTATGATCTGTGGGAGGT** | **200** | **KNP_645H8(F)** |  |
| **10** | **65870916** | **bE135G23SP6** | **TTTTCCGTTCAGAAAAAGAA** | **TGATTCTCCTGCCTACTTGT** | **193** | **KNP_645H8(F)** |  |
| **11** | **65965885** | **bT84I8SP6** | **GCTCTTCTCTTTGCTTTTCA** | **AGCATCTGTGCTTTCTTCTC** | **195** | **NP_738G12(F)** |  |
| **12** | **66012715** | **bT256E5SP6** | **TCCTTTGCCTTTACTTATGG** | **AAACAAAACCAGAAAGCTGA** | **171** | **NP_738G12(F)** |  |
| **13** | **66049417** | **bE209E6SP6** | **ATGCAGTAGCACCTCTAGGA** | **ATCTGTCAAGGCACTGTGAT** | **187** | **KNP_738G12(F), KNP_281D4(F)** |  |
| **14** | **66106471** | **bE90L15T7** | **AGAGTATCCAAGGAACAGAGG** | **GCACTCTATGGCTAATCACA** | **167** | **KNP_281D4(F)** |  |
| **15** | **66178215** | **bE19M5T7** | **ATAAGACATGCGTCTGCTCA** | **GTGCACAGCTGCCTTATCTA** | **165** | **KNP_281D4(F), KNP_1208B4(F)** |  |
| **16** | **66234859** | **bE176O22SP6** | **ACTCTAAATTCATGTTCTTTTCAG** | **AGTTTGGGGTCTGCTTCTA** | **198** | **KNP_1208B4(F), KNP_1462A5(F)** |  |
| **17** | **66287498** | **bE8P17T7** | **CAGTTATGAATGGGAAAAGC** | **AACCCTGAGATGGTCTTTCT** | **200** | **KNP_1462A5(F)** |  |
| **18** | **66352637** | **bE19M5SP6** | **GGGGAAATTATAGGTGGAAA** | **TCTGAAAAGGGGACTATGTG** | **200** | **KNP_1462A5(F), KNP_1137C9(F)** |  |
| **19** | **66419732** | **bT196K12SP6** | **TCAAAATAAACCCCAAAAGA** | **TTTAACGAATGCAGACCTTT** | **198** | **KNP_1137C9(F)** |  |
| **20** | **66469478** | **bT74A6SP6** | **ACAGAACACGGTACATCCAT** | **TCAATAATCCCATTTCCTTG** | **200** | **KNP_152B8(F)** |  |
| **21** | **66518296** | **bE2I22T7** | **CCATCTTAGGAGTCAGACCA** | **GCCACTTTACACTCTGATCTC** | **195** | **KNP_152B8(F), KNP_999A3(F)** |  |
| **22** | **66561489** | **bE170A8SP6** | **TATGACTTTCCAAGGTAGGC** | **AGACATGGTTTTGCTCACTT** | **196** | **KNP_999A3(F), KNP_1295F6(F)** |  |
| **23** | **66585088** | **bE68O6SP6** | **ACTTGGTTTTGCATTTTGTT** | **ACTGTTAAGGCCATCCTGTA** | **199** | **KNP_999A3(F), KNP_1295F6(F)** |  |
| **24** | **66766975** | **bE108P12T7** | **TCTCTTTCAGTGAGGGTGTT** | **AGACATGCCCTGATATGTTC** | **196** | **KNP_442E8(F), KNP_47E7(F)** | **CH242_428C20(U) )**** |
| **25** | **66816120** | **bT109A12T7** | **TTCAGCTTCAAGATGGATTT** | **ATATTCTCCAATCCCCAAGT** | **205** | **KNP_47E7(F)** | **CH242_428C20(U)** |
| **26** | **66865028** | **bE6A23SP6** | **CTCAGAGGACAAGGACAGAG** | **CCTTTAAGCCCCTTCTTTAG** | **196** | **not screened** | **CH242_428C20(U** |
| **27** | **66881151** | **bE280L20SP6** | **GCCTCTCTAAATATCCCTGAG** | **GGTCTGATGCTGTAGCTTTC** | **200** | **not screened** | **CH242_428C20(U)** |
| **28** | **66947508** | **bE275H22SP6** | **TTGCTACACGTTTTTCAGTG** | **AAGAACAAGTGGATGGAGAC** | **169** | **not screened** | **CH242_386F13(U)** |
| **29** | **66993458** | **bE118J17T7** | **AATACGTACCAAGAGCGAAA** | **CGTTTTAAATGGCTGCATA** | **192** | **not screened** | **CH242_271H15(U)** |
| **30** | **67074630** | **bE138L14SP6** | **TTAAGAAGGGAGACCTGTGA** | **TGGGATGTGAGAAGAGAATC** | **201** | **not screened** | **CH242_271H15(U), CH242_74A1(U)** |
| **31** | **67166151** | **bE271H15SP6** | **GGGAAAACTCCCTCTACATT** | **TCCTTAAGATGGCTCCTAAA** | **217** | **not screened** | **CH242_271H15(U), CH242_74A1(U)** |
| **32** | **67221743** | **bT111O19T7** | **GAATACTCGCACTACCCAAG** | **ATTATCTCCAGCAGCACATT** | **194** | **KNP_1661D10(U)** | **CH242_74A1(U), CH242_45N21(U)** |
| **33** | **67346148** | **bE258L18SP6** | **TCTTCCCTTTGTTTATCGTG** | **TCCTTTCTGCTTTCTAATCCT** | **207** | **not screened** | **CH242_45N21(U), CH242_253K6(U)** |
| **34** | **67421055** | **bE42B13T7** | **TGTCCCTGACAAAGTAACCT** | **CTTATTTCCTGGATCCCTCT** | **200** | **KNP_1459F12(F)** |  |
| **35** | **67474658** | **bT101F13T7** | **TTGCTGCCTAGAAGTCTCAT** | **TAAAAGCAAAAACCTTGGAG** | **200** | **KNP_1459F12(F)** |  |
| **36** | **67542418** | **bT105P21T7** | **CTTAACCGGTGTCCAATATC** | **ATGCTAGTGCAGTTTCCATT** | **200** | **KNP_320D2(F)** |  |
| **37** | **67607222** | **bT131L14T7** | **GGATGAATTTCCCTCTTCAT** | **TCATTGCCACACAGAAGATA** | **205** | **KNP_320D2(F), KNP_292F10(F)** |  |
| **38** | **67675029** | **bT241B22SP6** | **AAACAGGAAGCAACTTGAAA** | **TTAAAGGCTGAAGAAACAGC** | **202** | **KNP_222D11(F), KNP_292F10(F)** |  |
| **39** | **67794166** | **bT79I23SP6** | **TTGGTCATAATTGCCTTCTT** | **CTTTCTTCCCCAAAATATCA** | **195** | **KNP_222D11(F)** | **CH242_72N1(U)** |
| **40** | **67882709** | **bE96C2T7** | **CCTTAATCTCATGGAAGCTG** | **TGAGGTTCTGTCTTTCCATC** | **189** | **KNP_175E3(F)** | **CH242_72N1(U)** |
| **41** | **67955070** | **bT5F24SP6** | **AGGAAAGGATGAGGAGAAAG** | **ATCCTGTGCCTTTAGCAATA** | **194** | **KNP_175E3(F), KNP_807D1(U)** |  |
| **42** | **68010390** | **bE42O16T7** | **AATTTGGGGTCCATTAAACT** | **GCATTCAGGTTAAGAGGCTA** | **200** | **KNP_807D1(U)** | **CH242_462B18(U)** |
| **43** | **68070397** | **bE161O15SP6** | **TAGACCTTGTGGAGTTGGAC** | **TAGAGAACACGCAGACACAG** | **201** | **not screened** | **CH242_462B18(U)** |
| **44** | **68118720** | **bE246G7SP6** | **AATTCCTAACCCTGGATGAT** | **TGATTCACTGGTGTGATAGC** | **199** | **KNP_396G4(F)** |  |
| **45** | **68263559** | **bT133C5SP6** | **GAAACAGGAAGAGAGGTGTG** | **CCAGCTCCTTTGAATAACAC** | **192** | **KNP_561B9(F)** |  |
| **46** | **68343787** | **bE246G23T7** | **ACTATGAAACGATGGCAGAC** | **ATGTCCTTTCTGCATTTTCC** | **160** | **KNP_561B9(F)** |  |
| **47** | **68417032** | **bE20A9SP6** | **TCTCATGAGTATCCCTTTGG** | **CTCCATCTCTGTCTGCCTAC** | **201** | **not screened** | **CH242_497L3(U)** |
| **48** | **68558322** | **bT173G9SP6** | **TGAAAGCAAGATTTTTCCAT** | **CCCTGTGAAATACTCACCTC** | **184** | **not screened** | **CH242_497L3(U), CH242_22F14(U)** |
| **49** | **68621398** | **bT128F24T7** | **TCTGTAGAAGGGGGATAACA** | **AATGTAGGCAGTTGCTCTGT** | **203** | **KNP_553E4(F), 313E11(U)** | **CH242_22F14(U)** |
| **50** | **68673713** | **bE285E20SP6** | **TGCTGACATGCATTATCATT** | **TGGTGATGAAAAATGTCTGA** | **199** | **KNP_553E4(F), 313E11(U)** | **CH242_22F14(U), CH242_318C24(U)** |
| **51** | **68733439** | **bT253K17SP6** | **TGCCATGAGTTTTGTTTTG** | **TGCCGGTAATATTTATGTCAG** | **245** | **KNP_553E4(F)** | **CH242_318C24(U)** |
| **52** | **68804767** | **bT128F24SP6** | **AGCATATAGCCTCAGAGTTTTC** | **GTTATTTTTCTGCCTGTGGT** | **199** | **not screened** | **CH242_318C24(U), CH242_431A18(U)** |
| **53** | **68863507** | **bT260L9SP6** | **TATAGCTTCTTGGCCCTGT** | **TAAAACCAAAAACCCTCAAA** | **200** | **not screened** | **CH242_431A18(U)** |
| **54** | **68985541** | **bE97F18T7** | **AAGCAACACCTTTGTGAAGT** | **GCGAGGTCTGAGATGTAGAG** | **173** | **KNP_397E1(F)** | **CH242_237E14(U)** |
| **55** | **69055118** | **bE226H17T7** | **ATCAATTTAAGCCCCTCCT** | **GGGAAGTGTTTGTCTAGAGTG** | **184** | **KNP_397E1(F)** | **CH242_237E14(U)** |
| **56** | **69123351** | **bE169C13SP6** | **TAGGGAGATGCGACTAAATG** | **ATGTACATTCCTGCAAGGTC** | **198** | **KNP_397E1(F), KNP_1851G6(U)(2nd)** | **CH242_237E14(U)** |
| **57** | **69202841** | **bT177M13T7** | **AGACAGCAACTTTTGTCCAG** | **GAAGACATTAGCAGCCTCTG** | **200** | **KNP_1851G6(U)(2nd), KNP_76H6(U)** |  |
| **58** | **69271335** | **bE250J10SP6** | **AAGGCCTGTCTCTACCCTAT** | **CTTCTTTGTCTCAGGTTGCT** | **198** | **KNP_76H6(U), KNP_151C1(F)** |  |
| **59** | **69375383** | **bE169C13T7** | **AAGCAAAACTGATGCTGATT** | **TGTGAATGACAAAGATCACG** | **208** | **KNP_151C1(F)** |  |
| **60** | **69421076** | **bE110K23T7** | **AGGGATTTTGCTAGTTGCTT** | **GTGACCTGAGTCCACAATCT** | **195** | **KNP_151C1(F), KNP_1180D11(F)** |  |
| **61** | **69488157** | **bE78K12SP6** | **TGGAGAGAACAGGGTAGAAA** | **CCCATCTGAACTGACTTAGC** | **201** | **KNP_214F7(F)** |  |
| **62** | **69558879** | **bT199N16SP6** | **TGGTTTCTGTCCTTTCTTTG** | **GCATAGGATAATGAGGGCTA** | **198** | **KNP_214F7(F), KNP_1415H5(F)** |  |
| **63** | **69603534** | **bE133B5T7** | **AGGGTACATTGGTGACAAAG** | **ACAAGATGACTGGTGATGGT** | **212** | **KNP_1415H5(F), KNP_400G8(F)** |  |
| **64** | **69667115** | **bE92G15SP6** | **AAGAAATACTTTGTGCCATCA** | **TGTGATTCAAGACCATATTCC** | **198** | **KNP_400G8(F)** |  |
| **65** | **69736671** | **bE183E24SP6** | **CTTTGGTGTACAGAGGAGGA** | **TGGATTTCTAAGGACAGTGAG** | **175** | **KNP_400G8(F), KNP_1205C11(F)** |  |
| **66** | **69806961** | **bT60G2T7** | **AAAAGGACAGATGAGACCTG** | **GTTGAGCTGAGGTTGATTTT** | **200** | **KNP_1205C11(F), KNP_1532C12(F)** |  |
| **67** | **69861202** | **bE134N14T7** | **TCATAGGCTTTTCCAAGTGT** | **CAAGTTGGTTCTTACCCAGA** | **203** | **KNP_1532C12(F), KNP_1128D2(F)** |  |
| **68** | **69919865** | **bE2N4T7** | **TCAAGCAAACTTTCAGTAAAAT** | **AGGGGGTATGTAAATGGAGT** | **201** | **KNP_1128D2(F)** |  |
| **69** | **69987359** | **bT99M24T7** | **GCCACCATTCAGTACAAATC** | **AGTCTTCTCCAACTGTTCCA** | **169** | **KNP_1128D2(F), KNP_1755H12(F)** | **CH242_43M18(U)** |
| **70** | **70032394** | **bE191K4T7** | **AACAATGGGAGAGAAAATTG** | **CATTTGAAGGGAACGATAGA** | **202** | **KNP_1755H12(F), KNP_1553H6(U)** | **CH242_43M18(U)** |
| **71** | **70101831** | **bE2N4SP6** | **CTGCTCTAGGCAACAGAGAT** | **AATTCTTCCAGAGACCCTTT** | **196** | **KNP_309H8(U)** | **CH242_43M18(U)** |
| **72** | **70156600** | **bT243K9SP6** | **CATGTTCATGTGCTAGTTGC** | **GCACAAAGTGAATGAATGAA** | **196** | **KNP_309H8(U), KNP_1145F5(U)** |  |
| **73** | **70187934** | **bT140J14T7** | **GAAGTCAGAATTGCTTGGTT** | **TTTTAATGATGCAATGACCA** | **196** | **KNP_309H8(U), KNP_1145F5(U), KNP_1231E9(F)** |  |
| **74** | **70247379** | **bE80K5SP6** | **ATATCACTGGGTGCTAGGTG** | **CCTCTTGCTGAGTACAGGAC** | **200** | **KNP_1231E9(F)** |  |
| **75** | **70271765** | **bT137F18SP6** | **TAGTCCTCTACCTGGGTGAA** | **CACACTTGTATCCCCAAACT** | **198** | **KNP_1231E9(F), KNP_1176A2(F)** |  |
| **76** | **70332934** | **bE185D13T7** | **ACAAATCAAATGCTGTGTGA** | **CAAATTTGAAAATGCACAAG** | **210** | **KNP_1176A2(F)** |  |
| **77** | **70387277** | **bE113G2SP6** | **ATTTTCAACAACCAAAAACG** | **TTCTAGTTGAGGATGCAGGT** | **194** | **KNP_1176A2(F)** |  |
| **78** | **70444048** | **bE226H23T7** | **AGCGTGTAAGCTACAAAAGC** | **GCCAGCATACGAGATAGTTT** | **202** | **not screened** | **CH242_516C3(U)** |
| **79** | **70500273** | **bE226I14T7** | **AACAAATCACAGCACTCACA** | **GAAAAGGGAATCATGTTGAA** | **195** | **KNP_1842D11(F)** | **CH242_516C3(U)** |
| **80** | **70551020** | **bE208H2SP6** | **AAGGAATTTTCAGAGGGAAG** | **GAAAGAGTGTGCAACCAAAT** | **212** | **KNP_1842D11(F)** | **CH242_516C3(U)** |
| **81** | **70655264** | **bE259C8SP6** | **TAGGAGTCAGGTTTCCTTGA** | **CTCCAATTCCTACACTCCTG** | **204** | **KNP_1682F12(F)** |  |
| **82** | **70685229** | **bE227M22T7** | **GATATTGAAATGGAGGGAAA** | **CCATTGTTCTGCTTCTTTG** | **161** | **KNP_1682F12(F), KNP_824C4(F)** |  |
| **83** | **70790265** | **bE43B23SP6** | **TGAATTCAAATTGTCTGGAA** | **GTTTACCCAAGTAGGGGTTT** | **182** | **KNP_1682F12(F), KNP_824C4(F)** | **CH242_392P23(U)** |
| **84** | **70841322** | **bT201O4T7** | **GCCAGTATGTTGCCAGTAAT** | **TGGATGAAATCAAAGGAAGT** | **203** | **KNP_440B2(F)** | **CH242_392P23(U)** |
| **85** | **70906587** | **bT142H17SP6** | **GAAAAGGAGGTTTTTCTGGT** | **TGGCTATCAGGCTACCTTTA** | **200** | **KNP_440B2(F), KNP_453H8(F)** | **CH242_392P23(U)** |
| **86** | **70976195** | **bE233E8T7** | **CAGGTATGCAGGCAGTTATT** | **CCACTAGTGCATGTGTGTGT** | **169** | **KNP_440B2(F), KNP_453H8(F), KNP_679G9(F)** |  |
| **87** | **71047425** | **bT1L14T7** | **CAGTGTCCTCCAAATTTCAT** | **GAACTGTGTTCTGGGATAGG** | **200** | **KNP_453H8(F), KNP_679G9(F)** |  |
| **88** | **71079637** | **bE53H16T7** | **GGCAGAAGAATAGATGCCTA** | **ATCATCTTTGCCATTCAGTT** | **202** | **KNP_679G9(F)** |  |
| **89** | **71121436** | **bT144B18T7** | **CATGCAAGCTTGTTAAGTCA** | **CACTTGAAACCCTTGTTTTT** | **208** | **KNP_679G9(F)** |  |
| **90** | **71165671** | **bE86L8SP6** | **ACTCCGTTTCCTACATTCAA** | **CAGAGCCCATAATTCTCTTG** | **200** | **not screened** | **CH242_60I9(U)** |
| **91** | **71220389** | **bE165C9T7** | **TTACATACTGCCTCCCACTT** | **CAAGAATGGCTTCACTAACC** | **195** | **not screened** | **CH242_60I9(U)** |
| **92** | **71275789** | **bE186J17SP6** | **AAAGATATGATGGCATTTGG** | **TATGAAACAGAGGCAGAGGT** | **198** | **KNP_1808G5(U)** |  |
| **93** | **71332065** | **bE86L8T7** | **TGCTCCTAAAGACCAGAAAA** | **GGAGCTTTTTAATGAACGTG** | **197** | **KNP_1808G5(U), KNP_174C12(F)** |  |
| **94** | **71403780** | **bT132K19T7** | **CCTGGTCATCCTCTATTCAG** | **GGATGAGAACAGAGGTGGTA** | **201** | **KNP_174C12(F)** |  |
| **95** | **71465910** | **bE191N20SP6** | **ACAAAGTGGAAGGGATCTTT** | **ATTGATGTGAATGAGGAAGG** | **198** | **KNP_174C12(F)** | **CH242_372K6(U)** |
| **96** | **71518450** | **bT3F13T7** | **GGAATTTTAACCCCAGTTCT** | **ATCCATTTGACTATCCCTTT** | **179** | **KNP_1024E7(F)** | **CH242_372K6(U)** |
| **97** | **71600360** | **bT60C15SP6** | **CTGTCTCCATTGCACTTGTA** | **AATTAAAAGATCAGGCAGCA** | **197** | **KNP_1024E7(F)** |  |
| **98** | **71663636** | **bT149I1T7** | **CCAGAAAGAACTCCAAACAG** | **CATTGATCTCCTTTCATGCT** | **198** | **KNP_283F4(F)** |  |
| **99** | **71712070** | **bT261A24SP6** | **TCTGTAATGCACACGAATGT** | **TGGTTTCTTTCAAGTCATCC** | **203** | **KNP_283F4(F), KNP_1056G6(F)** | **CH242_269D21(U)** |
| **100** | **71780368** | **bT87I10SP6** | **TGTAAGCAAGACTGGCAATA** | **TGGACAAAGCACATAATTGA** | **206** | **KNP_1056G6(F), KNP_797D2(F)** | **CH242_269D21(U)** |
| **101** | **71841713** | **bT261A24T7** | **TCCTTTGGACTTACTGCTCT** | **TTCTACATGGTTGAGCACTG** | **198** | **KNP_797D2(F)** | **CH242_269D21(U)** |
| **102** | **71861691** | **bT242J9T7** | **TTGAGGGGCACTAAATAAGA** | **AAGGCAATTTGACAACTGAT** | **202** | **KNP_797D2(F)** |  |
| **103** | **71973156** | **bE233I8SP6** | **ATAACTTGCTGACTCCCAAA** | **TTGAAGTGTGTATTGGCAGA** | **199** | **KNP_299A4(U)** | **CH242_197D10(U)** |
| **104** | **72014460** | **bT196K21T7** | **TTGAACACATCTTTCACAGC** | **AATAGCAGCACGGTTACACT** | **197** | **not screened** | **CH242_197D10(U)** |
| **105** | **72068179** | **bE115L6SP6** | **AAGAACATCAACCCATCAAA** | **TGCATGAGTCAGAGATGAAA** | **196** | **not screened** | **CH242_197D10(U), CH242_210I22(F)** |
| **106** | **72126021** | **bE180O9T7** | **CTGGCTTGCTTTGGTACTAT** | **CTTGGTCCCAAATACTGTGT** | **199** | **KNP_349C1(F)** | **CH242_210I22(F)** |
| **107** | **72179073** | **bT240L24T7** | **GTAAGCCTTAGCACCATCAC** | **CTCCCGTCATAGGAATATCA** | **194** | **not screened** | **CH242_210I22(F)** |
| **108** | **72227750** | **bE8F6T7** | **ACCACAAGCCTCTACTTTCA** | **ACAAGGAGTTTTGGTTGAGA** | **201** | **not screened** | **CH242_210I22(F), CH242_328M12(U)** |
| **109** | **72262065** | **bE259E16T7** | **CCTATCAATCACCTCGTCAT** | **CATGTAGTCAGATGGCTGAA** | **207** | **not screened** | **CH242_328M12(U)** |
| **110** | **72339500** | **bE35N4T7** | **TTTGACTCTGCCTTTCTCTC** | **TTTGTGGTAAATGCTGAGAA** | **197** | **not screened** | **CH242_328M12(U), CH242_225O20(U)** |
| **111** | **72404170** | **bT196F8SP6** | **CATAGGATTTCCTTCCCATT** | **ATGTGTGCCTCATTAATTCC** | **197** | **not screened** | **CH242_225O20(U)** |
| **112** | **72501231** | **bE178F11SP6** | **TCAAAATTCAACTCACAGGA** | **TGATCACTTTTGATCAGCAT** | **202** | **KNP_456G10(U)** | **CH242_225O20(U)** |
| **113** | **72646694** | **bE105I22SP6** | **TTTGTCAATGTACAGGCAGA** | **TTGCTGCAAAAAGTAAAACA** | **199** | **KNP_86D3(U)** | **CH242_272F18(U)** |
| **114** | **72701669** | **bT228A10SP6** | **CATGTCTTCCAAGGTTTAGG** | **CTGTCTCACCTCCTTTCTTG** | **206** | **KNP_828H7(F), KNP_1770C10(F)** | **CH242_272F18(U)** |
| **115** | **72774780** | **bE98P11SP6** | **AGTCATCTGGCTCATCCTAA** | **ACATTCTTGGGGGTAGAAAT** | **197** | **KNP_828H7(F), KNP_1770C10(F)** |  |
| **116** | **72842970** | **bT210B14SP6** | **TAAGCCAATTTTCTGGGTAA** | **CTGCTTTGTGTTTGTGATGT** | **202** | **KNP_1216C2(F)** |  |
| **117** | **72881453** | **bE164K22SP6** | **AAAAATATTAGGGCCGAAGT** | **ACTCTTTTCCCCCAAATAAC** | **194** | **KNP_1216C2(F)** |  |
| **118** | **72944744** | **bT85B1T7** | **CAAGCTTGCTAAACCAGAAT** | **TTAAAACTAGGGCAAATGCT** | **195** | **not screened** | **CH242_249B5(U)** |
| **119** | **72984265** | **bE203A20T7** | **TAAGAACACAGCCTTGGTTT** | **AATGTGCACTTAAAGGGAAA** | **193** | **not screened** | **CH242_249B5(U)** |
| **120** | **73017910** | **bT210B14T7** | **TTTTCCCTCAATAAGCATGT** | **CAGAATACATCGTCAGCAAA** | **200** | **not screened** | **CH242_249B5(U)** |
| **121** | **73077249** | **bE44D15SP6** | **TCACCGTATGGCTTATTTCT** | **GATGGAAGCAACAGTACACA** | **200** | **KNP_1845F5(F)** | **CH242_249B5(U)** |
| **122** | **73134623** | **bT144I22T7** | **GGCTCAACAAGAGTATCAGC** | **ATAGGATAGGCCCAGAAAAC** | **198** | **KNP_1845F5(F)** | **CH242_249B5(U)** |
| **123** | **73173134** | **bT238P19SP6** | **GTTTCCTGATTCCCTAAACC** | **AATCAAAGAAATGGGAGGAG** | **220** | **not screened** | **CH242_249B5(U), CH242_219M19(U)** |
| **124** | **73236786** | **bE115P24T7** | **ATAACGCCCTTCCTTTTACT** | **ACTGTTAATTTTTGCGCATT** | **200** | **KNP_142D10(F)** | **CH242_219M19(U)** |
| **125** | **73279522** | **bT106D24T7** | **CTCAGCTTGACACACGACTA** | **AAACACACACACCATTTTCA** | **200** | **KNP_142D10(F), KNP_1293H1(F)** |  |
| **126** | **73399922** | **bE30J20SP6** | **GGAAGAACAAAATGGTGATG** | **ATTCCCATCCCTAAGAGAAA** | **200** | **KNP_1293H1(F)** |  |
| **127** | **73424462** | **bE265B7T7** | **CAATTGCCTCAAAATTAAGC** | **ACACCATTTAGAGTGCCTCA** | **200** | **not screened** | **CH242_73A23(F)** |
| **128** | **73536533** | **bT135I13SP6** | **GTGTGGATACACCTCCATTC** | **ACTCCTCTATTCCAGGCACT** | **192** | **KNP_1069E7(F)** | **CH242_73A23(F), CH242_173F13(U)** |
| **129** | **73588933** | **bT197A22SP6** | **ATTTAGAGCAGGCCATCATA** | **ATCTGCAGCCATCATACTTC** | **168** | **not screened** | **CH242_173F13(U)** |
| **130** | **73682233** | **bT135I13T7** | **AATGTGAAAGCAAAGCAACT** | **CCATACATGGCTGAAAGAGT** | **200** | **KNP_1040B11(F)** | **CH242_173F13(U)** |
| **131** | **73722656** | **bE213L2SP6** | **TGAAGGATCCCTGAATAAGA** | **GTCTGTAAAATGGGCGTAAC** | **199** | **KNP_1040B11(F), KNP1228B3(U)** |  |
| **132** | **73938689** | **bE213L2T7** | **CAATTTCCGGCTATAATCTG** | **CCCCTGAAACATTATAAATCA** | **202** | **KNP_277G11(F)** | **CH242_194O23(U)** |
| **133** | **73996954** | **bT148N13T7** | **GAGAAATCAAGGCAATGAGA** | **AGGAACAATGCTTCTCAAAA** | **196** | **KNP_277G11(F)** | **CH242_194O23(U)** |
| **134** | **74062386** | **bT148N13SP6** | **CCTCTTTCTCCCAGATTCTT** | **GTGGCCACAGTCTTACAGAT** | **201** | **KNP_1461D9(F)** | **CH242_508K2(U)** |
| **135** | **74115936** | **bE133C16T7** | **AGCCTTTTGCCTGTGTACTA** | **AGAGCGAAGGACTCTTTTCT** | **198** | **KNP_12G3(F)** | **CH242_508K2(U)** |
| **136** | **74145755** | **bE201F17SP6** | **TTCCTGATGAGTTTGTCACTT** | **TGCCTAAGAATCAGAGGAAA** | **202** | **KNP_12G3(F)** | **CH242_508K2(U)** |
| **137** | **74235326** | **bT253H2SP6** | **TTTTGGAAAATCATCAGGTC** | **CAGTGCTGATCACTCAGGTA** | **193** | **KNP_736F12(F)** |  |
| **138** | **74279464** | **KNP_736F12** | **GCAGGAGAGCCATAAAAATA** | **TTTTTCCTTCCAGAAACAGA** | **230** | **KNP_736F12(F), KNP_139H12(F)** |  |
| **139** | **74320011** | **bE259L13SP6** | **TACACCAACATGTGAATGCT** | **TATGCATTGAGTGCCAACTA** | **201** | **not screened** | **CH242_199D6(U)** |
| **140** | **74420710** | **bE199A18SP6** | **ATTCAGAACTGGCATCTTCC** | **AGGTACATAATTTAAAAGCTAAAA** | **155** | **KNP_976E4(F), KNP_1249H6(F)** | **CH242_199D6(U), CH242_24J21(U)** |
| **141** | **74483009** | **bE215K17SP6** | **TATGGGGAAGCTATTTTCAA** | **CCAGAGGTGGAACACATAGT** | **201** | **KNP_976E4(F), KNP_1249H6(F), KNP_1327B3(F)** | **CH242_199D6(U), CH242_24J21(U)** |
| **142** | **74539801** | **bT100G10T7** | **TCATTGAGTGCTCAGTCGT** | **CAAGTCCTCCAAACAGAACT** | **190** | **KNP_1327B3(F)** | **CH242_24J21(U)** |
| **143** | **74561728** | **bE24J21SP6** | **TCCACCCTATTTAGCCACTA** | **TAGCTTTTCACAAAGCACAC** | **195** | **not screened** | **CH242_24J21(U), CH242_259G13(U)** |
| **144** | **74618313** | **bE226O21SP6** | **TCACAGAATTGCTGTTTTTG** | **CACCTCTCGATGTAACTTCC** | **198** | **not screened** | **CH242_259G13(U)** |
| **145** | **74675422** | **bT146M9SP6** | **TCTGACTATTCCCCAGACAC** | **GTGCAAACAAAGTGCTACAA** | **205** | **not screened** | **CH242_259G13(U)** |
| **146** | **74709632** | **bT124F15T7** | **CTCCCTCGTAAGGAATTTTT** | **GGTCCTCATGGACTGTTAAA** | **195** | **not screened** | **CH242_259G13(U)** |
| **147** | **74817574** | **bT146M9T7** | **GAGTAGGCAGCACCTAACTG** | **AGCAGCTACTGAACCAAAAG** | **206** | **KNP_1812D2(F)** | **CH242_163B16(U)** |
| **148** | **74878695** | **bT121L9T7** | **GAACAAATCCAGAGGTCAAA** | **CAAATACCTTCACTGGCTCT** | **200** | **KNP_1812D2(F)** | **CH242_163B16(U)** |
| **149** | **74934570** | **bT179D9SP6** | **ACAGGTGCTAATTAGGAGGA** | **GTGTGTGTATGCGTAGCTTG** | **205** | **KNP_431E6(F), KNP_1812D2(F)** |  |
| **150** | **75018674** | **bE247G9SP6** | **GAGCAAAACATTCCATAAGC** | **AATTAACCAACCAAACAAAAA** | **200** | **KNP_431E6(F)** |  |
| **151** | **75070745** | **bT219G15SP6** | **AGGCCTTCATTTTACCTTTC** | **TCCTGCTTACCTTTACCTCTT** | **199** | **not screened** | **CH242_247G9(U)** |
| **152** | **75133075** | **bE224P7T7** | **AATGCAGACTGGTTTCCTAA** | **CATGGCTAGCACTAACAACA** | **203** | **KNP_1491C11(F)** | **CH242_247G9(U)** |
| **153** | **75183294** | **bT91P4T7** | **CCTGAAAGCAATAGATTTGG** | **GTTGGTATTATGGGCACAGT** | **200** | **KNP_1491C11(F)** |  |
| **154** | **75236835** | **bE257L2T7** | **TAGTGACGTTGCACCTGATA** | **TCCTGTGGAATCATATAGGG** | **205** | **KNP_1491C11(F), KNP_1257H5(U)** |  |
| **155** | **75310384** | **bT211I23SP6** | **GCTGGTACAACCTCTTTGAA** | **TGCTCCACTAGTGTCCTTTT** | **205** | **KNP_1257H5(U), KNP_1F2(F)** |  |
| **156** | **75363249** | **bE153E7T7** | **GTGATTCCAGGAGACAAATG** | **CACATCTCTGATCCAAGCTC** | **205** | **KNP_1257H5(U), KNP_1F2(F)** |  |
| **157** | **75431798** | **bT251E2SP6** | **TTCACCATAAAACCTCAACC** | **GGCAAAGTATAGTGCCTGAG** | **168** | **KNP_1F2(F), KNP_928A4(F)** |  |
| **158** | **75489276** | **bE235I21T7** | **ACAGCTGAGCAGGTTAAAGT** | **TGTTAGGCAGCTTAAAGAGG** | **195** | **KNP_928A4(F), KNP_1144E6(F)** | **CH242_293N21(U)** |
| **159** | **75561871** | **bT119J23SP6** | **TTATGCGCCTAGAGTTCTTC** | **TCTCTCAAAGGTTCTCCTCA** | **202** | **KNP_1144E6(F)** | **CH242_293N21(U), CH242_419P15(U)** |
| **160** | **75659403** | **bE180G17T7** | **GCTTTAAAATGGGAACAGGT** | **CCTTTACTTCCTCCTCAGGT** | **200** | **not screened** | **CH242_419P15(U)** |
| **161** | **75727401** | **bE178E20T7** | **AGAAGCCTTCATCATTGAAA** | **CATTCCACTCTTTTGGTTGT** | **204** | **KNP_1699H3(F)** | **CH242_419P15(U)** |
| **162** | **75795687** | **bE163E10T7** | **GGTCATCATTTTCCATGTCT** | **AGCACGGCACTATAGTAAGC** | **205** | **KNP_1699H3(F), KNP_706C6(F)** |  |
| **163** | **75853181** | **bE199K12T7** | **GTTCTGAATCCATTTTCCAA** | **CCCTGAACTTTCCAACTTTA** | **196** | **KNP_706C6(F)** |  |
| **164** | **76024216** | **bT219A8SP6** | **ATGTGGTCACCTAAGTCTGG** | **ACCGGTGACTAAAATGCTTA** | **199** | **KNP_905B10(U), KNP_1652F10(F)** |  |
| **165** | **76054597** | **bE38E22T7** | **TTACAGGTATTACTGCCTTGC** | **AAAAATTGGATGATCTTACCT** | **199** | **KNP_1652F10(F), KNP_905B10(U)** |  |
| **166** | **76122490** | **bT232J13SP6** | **ATTACAACACTGGGCAGAGA** | **ATTCTCAAGGAACCCAGTTT** | **203** | **KNP_1652F10(F)** |  |
| **167** | **76172000** | **bE92H13T7** | **GCAAAAGGAAAAAGAAAACA** | **TCGTCTCTTTATTAGGCAGAA** | **216** | **KNP_1521E8(F), KNP_188E3(F), KNP_994C10(F)** |  |
| **168** | **76229879** | **bT116B9T7** | **TCTTTCTCAAAAGCCACTTC** | **GGGCATCAATTAGAAGTCTG** | **199** | **KNP_1521E8(F), KNP_188E3(F), KNP_994C10(F)** | **CH242_18D11(U)** |
| **169** | **76290871** | **bE98M7SP6** | **CAGAATACATTCCGGTTCTC** | **TCTTTCTGTGGAAAATGACA** | **190** | **KNP_1752F12(F)** | **CH242_18D11(U)** |
| **170** | **76321496** | **bE153C9SP6** | **AATAACTGTGCTGGGACAAG** | **GCCCTTCTCTTCTAGCTACC** | **198** | **KNP_1752F12(F), KNP_1287D5(F),** | **CH242_18D11(U)** |
| **171** | **76383032** | **bT116B9SP6** | **AAGAGGGGGATTCTTAGATG** | **CTGGGTTTGAATGTTAGCTC** | **199** | **KNP_1752F12(F), KNP_1287D5(F),** |  |
| **172** | **76450320** | **bE74P13T7** | **GCTGCGCAATAATTAGACTT** | **GCTCTTAGAGAATGCTGCTT** | **199** | **not screened** | **CH242_45N21(U)** |
| **173** | **76508226** | **bE153C9T7** | **CTTTTGGATCAGTCGCTTAC** | **GGTGTGTGAAATGGAAAAAT** | **196** | **KNP_439A4(F)** |  |
| **174** | **76561614** | **bT253G15SP6** | **GCCATCATCTCTGTTAGTCC** | **AAGTGTGGTCAATGAGCTTT** | **210** | **KNP_439A4(F)** |  |
| **175** | **76625231** | **bE257N2SP6** | **GGGAAGAAGACCAGAGAAAC** | **GAGGTTTTGAAGCGATCTAA** | **196** | **KNP_1487C3(F), KNP_957F3(U)** |  |
| **176** | **76663826** | **bT46D12T7** | **CTTGTAGACAGCACAGGTGA** | **AATGAAAGCAGCTGAGAAAC** | **195** | **KNP_1487C3(F), KNP_957F3(U), KNP_57C8(F)** |  |
| **177** | **76734820** | **bE88L11SP6** | **ACCTCCAGAGGTTTCTTTTC** | **TGGAGAGGTCATTTGTTTCT** | **201** | **KNP_57C8(F)** |  |
| **178** | **76785078** | **bE259N10T7** | **AGAAATGCGTTCACAAGAGT** | **TACATTCGAGGGGTATTCTG** | **195** | **KNP_1454H10(F)** |  |
| **179** | **76842297** | **bT122J13SP6** | **AAGGAATCTAGCATTGAACG** | **GTGAAGAGAGGCAGAGCTTA** | **200** | **KNP_1454H10(F), KNP_1001D9(U)** |  |
| **180** | **76902030** | **bE88L11T7** | **TTTCCTCAAACAGGTGGTAG** | **GACAACAGCCACTTCAATTT** | **208** | **KNP_1001D9(U)** |  |
| **181** | **76958682** | **bE220N9T7** | **GGGAGGAGGATATAGGAAGA** | **GCAGACTTGATGAGGAAGTT** | **206** | **KNP_1001D9(U), KNP_1519D9(U), KNP_309H8(F)** | **CH242_75I15(U)** |
| **182** | **77010349** | **bT100I4T7** | **ATCATCTGAGTCAGGAATGC** | **GAATTATGGAGGCTTGTGAG** | **206** | **KNP_1519D9(U), KNP_309H8(F)** | **CH242_75I15(U)** |
| **183** | **77060035** | **bT180O5SP6** | **GGGTGTCTCTGTTTCTTTGA** | **TAAAACTTGGTGGCTAGCAT** | **203** | **KNP_821F11(F)** | **CH242_75I15(U)** |
| **184** | **77111538** | **bE75I5SP6** | **GGAGACTAGAGCCTGGAGTT** | **ATAGCCCCTTTCTCAACAGT** | **197** | **KNP_821F11(F)** | **CH242_75I15(U)** |
| **185** | **77163723** | **bT93D2SP6** | **CACAGGAGGGATTTCAGTAA** | **ACTCTGCATGTTTTGCTCTT** | **201** | **KNP_821F11(F)** |  |
| **186** | **77234870** | **bE62E5T7** | **GAGTCCTGTGAAGTGCAAAT** | **TTGTCTCCAGGAAACCATAC** | **199** | **KNP_3E11(F)** |  |
| **187** | **77267131** | **bE110E1T7** | **ATGCAGACTTCCCTCTGTAA** | **TCATTTTGAGCCATAACACA** | **209** | **KNP_3E11(F)** |  |
| **188** | **77398870** | **bE62E5SP6** | **TTTCCTGAATTTTCTGCACT** | **GCCAGTCACTTAGCAAAAAT** | **199** | **KNP_1286A7(U)** |  |
| **189** | **77499536** | **bT242G3SP6** | **AACCAAAAGCAGGTAATGAA** | **AGGACCAAGACTATTGAGCA** | **200** | **KNP_881H4(F)** |  |
| **190** | **77528414** | **bE27E13T7** | **TTCCATGAAGTCAGTCAGTG** | **TTTCCTAAAGCATCCATTGT** | **202** | **KNP_881H4(F)** |  |
| **191** | **77596785** | **bE82N9T7** | **GACCCAGAAAAGACTCAACA** | **TTAAAACTGGAAGAGCCAAG** | **201** | **KNP_881H4(F),KNP_1212G8(F)** |  |
| **192** | **77650895** | **bE96B19SP6** | **TTGGCTTGTAACTGTGAGTG** | **GAAAAGCATATGGGAGCTG** | **172** | **KNP_1255H1(F)** |  |
| **193** | **77773533** | **bT98L18T7** | **ATCTGGTGGAAGCAAGATAA** | **CAGTCAGAGAGTGGCTAAGG** | **208** | **KNP_1260D5(F)** |  |
| **194** | **77854222** | **bE96B19T7** | **TTTTACAATGGCAGGGATAG** | **TCAAAGACCTCCTCGAATTA** | **199** | **KNP_1265C9(F)** |  |
| **195** | **77948507** | **bT246L19T7** | **GCTCCAATACCTTTGACAAC** | **GCAGTGAAGACCAAGAAGAC** | **195** | **KNP_1798G2(F),KNP_1545D7(U)** | **CH242_72B15(U)** |
| **196** | **78006334** | **bT166J11T7** | **CATGCAAGCTTCTCTAACCT** | **TTGGAAAAGCTGAAGAAATC** | **195** | **KNP_1545D7(U)** | **CH242_72B15(U)** |
| **197** | **78064249** | **bT70B15SP6** | **ACATAGGCACATGGTAAACAC** | **GTGTCAAGAGCACAACTGAA** | **173** | **KNP_619A9(F)** |  |
| **198** | **78141035** | **bT81M15T7** | **TGGTTAAAGAAGTGGCCTAA** | **CTGGACTATCACTCCAGCTC** | **200** | **KNP_619A9(F)** |  |
| **199** | **78210509** | **bT126D11T7** | **GAAGTTCAAAGAGTGCTGCT** | **CCTTCAGTTTTCAAGCAATC** | **198** | **KNP_1219A4(F)** |  |
| **200** | **78273681** | **bT44I4T7** | **ATTCGATATGAATGGCTTGT** | **TGTGCACAAAGTAGGTGGTA** | **213** | **KNP_1219A4(F), KNP_964E3(F)** | **CH242_426H1(U)** |
| **201** | **78287789** | **bT81M15SP6** | **TAAATCTGCCTGGTTCAAGT** | **GCAAGAGGAGTAACGGTGTA** | **203** | **KNP_964E3(F)** | **CH242_426H1(U)** |
| **202** | **78396337** | **bE28D10T7** | **TAGCACTAACCCTTTGCACT** | **GGTCTGAGTTGGAGAAATGA** | **205** | **KNP_964D7(F)** | **CH242_426H1(U)** |
| **203** | **78468069** | **bE270H17T7** | **CCCCACATTTTGTTAATCAG** | **TTCTTTTGGTCAACCCATAC** | **197** | **KNP_964D7(F)** |  |
| **204** | **78522697** | **bT73D8T7** | **GGGGTTACAAATACCATGAA** | **ATGAATTTGGGGATTAACCT** | **198** | **not screened** | **CH242_365O3(U)** |
| **205** | **78589371** | **bE194J9SP6** | **ATCAATGACCCAGAATTGAG** | **CATCTGGTAGCAGTCCAAAT** | **200** | **not screened** | **CH242_365O3(U)** |
| **206** | **78659884** | **bE265A16T7** | **CGTTCAACACATCAGAAAAA** | **TCTACTCTGTGCCCTCACTT** | **206** | **not screened** | **CH242_365O3(U)** |
| **207** | **78681988** | **bE395B2SP6** | **TCAATGAGCTCCCAATTTAT** | **ATGCAATTTTCCGTGAATAC** | **198** | **not screened** | **CH242_365O3(U), CH242_106J21(U)** |
| **208** | **78721015** | **bE14J5T7** | **GCGAGTCCATTTCTCAGTAG** | **CAAATCTGATCCCCTATTCA** | **187** | **not screened** | **CH242_106J21(U)** |
| **209** | **78775237** | **bT218N13T7** | **TTCTCTCTGAAACGTCTGGT** | **GAAATGACAACCCAAGGTAA** | **206** | **not screened** | **CH242_106J21(U)** |
| **210** | **78866121** | **bE50B12T7** | **CATGGAAATAGCTTTGGAAC** | **GCAGCTGTCATTTCCATTAT** | **197** | **not screened** | **CH242_106J21(U), CH242_375H16(U)** |
| **211** | **78927440** | **bT96G17SP6** | **ATTTGCCCTTGACAGTGTAT** | **TAAAATCCGGCAGGTATAGA** | **200** | **KNP_1681H1(F)** | **CH242_375H16(U)** |
| **212** | **78975154** | **bE262H23SP6** | **GTCCTATTTCTGATGGTCACA** | **AATGTGCTCTTTGCTGATCT** | **193** | **KNP_1681H1(F)** |  |
| **213** | **79026456** | **bT198F9SP6** | **CATTATTTGATCTGGGGAAA** | **CATGTGGAGGAATTATGGAT** | **198** | **KNP_1681H1(F), KNP_327G6(F)** |  |
| **214** | **79081784** | **bT96G17T7** | **TCCAGTCTTGTTTCCTCTGT** | **TGGTGTTTGAAATAGGCTTT** | **193** | **KNP_327G6(F)** |  |
| **215** | **79148476** | **bT173F1SP6** | **TTGTATGGCTCAAACTTTCC** | **CAGCAACATATTCTGCATTG** | **196** | **KNP_327G6(F), KNP_22D11(F)** |  |
| **216** | **79203584** | **bE108N2SP6** | **AGGAACTTTCAGATGCCATA** | **TATTCCTGGAGATACCCAAA** | **181** | **KNP_22D11(F), KNP_129H10(U)** | **CH242_513D23(U)** |
| **217** | **79260682** | **bE203G13SP6** | **TTTGCTAAGTGTGCATCAAG** | **GCAGGCAGGCTATAAAATAA** | **203** | **KNP_22D11(F), KNP_129H10(U)** | **CH242_513D23(U)** |
| **218** | **79422240** | **bE285M2T7** | **ATAGATCCTCCACACCACAG** | **ACTGATTGTTAATGCGGAGT** | **199** | **KNP_1035B4(F)** | **CH242_125O3(F)** |
| **219** | **79498569** | **bT260N7T7** | **GTCTGTGGAGAGATTTTTGG** | **CAGTGTCTCTTTCCATTGGT** | **200** | **KNP_1035B4(F), KNP_1098A3(U)** | **CH242_125O3(F)** |
| **220** | **79566835** | **bE125O3SP6** | **AACCATGATTGGTCAAAATC** | **GTGCTCCTTAAAAAGCAAAA** | **196** | **KNP_1098A3(U)** | **CH242_125O3(F)** |
| **221** | **79621067** | **bT224I11SP6** | **GAAGAGAGTTCGAAGGCATA** | **ATCCTTGTTGGAATTCACTG** | **200** | **KNP_266D9(F)** |  |
| **222** | **79735203** | **bE126I19SP6** | **GAGCTGTTGCCTATTAGCAT** | **GAATTGTACCAACTGGAAGC** | **201** | **KNP_266D9(F), KNP_893F3(F)** |  |
| **223** | **79783400** | **bE149N14T7** | **AAACCACAGTCCACACAAAT** | **TAATCTCAAAAGCCACAACC** | **197** | **KNP_893F3(F)** |  |
| **224** | **79828998** | **bE232H3T7** | **TTCGTCGAGGAGCTTAAATA** | **TCTGGATATTGTCCTTGGAG** | **199** | **KNP_893F3(F), KNP_347G6(F)** |  |
| **225** | **79872835** | **bE194H16SP6** | **AAGGCAGTATAAGCCACAGA** | **TGCTGAAAGTGATATGAACG** | **190** | **KNP_347G6(F)** |  |
| **226** | **79945998** | **bE96B18T7** | **ATTTGCCTCCAATATCAATG** | **TGCTCTCTGTGGGCTAGTAT** | **204** | **KNP_347G6(F), KNP_1462F11(F)** |  |
| **227** | **80011157** | **bE260P8T7** | **TTGAGCCTTTTAACCACATT** | **AGACTGCACTCATCCAGAAC** | **213** | **KNP_1462F11(F)** |  |
| **228** | **80080372** | **bT190M13T7** | **TTGTCAGCATATCCAAGTGA** | **TCCCCAAGATTGTAGTCATA** | **196** | **not screened** | **BAC clone gap** |
| **229** | **80231671** | **bT44A15SP6** | **GCTGCGAATGTAAAACAGAT** | **ATATCTGGGTCATCCTCCTT** | **211** | **KNP_26F12(F)** |  |
| **230** | **80426150** | **bE225E23SP6** | **CCAAGGGATAGAGACATCAG** | **CCTGTCAGCACTTACCTTTC** | **208** | **KNP_1020E1(F), KNP_1286D8(F), KNP_1731F8(F)** |  |
| **231** | **80484043** | **bT117E15SP6** | **GAAGGTATGGATGCACTGTT** | **TCACACTAGGCATGTCAGAA** | **190** | **KNP_1020E1(F), KNP_1286D8(F), KNP_1731F8(F)** |  |
| **232** | **80520571** | **bE140E24T7** | **GACAACCACCTTCAGATCAT** | **GTTTCAGGGACAGTTCTTTG** | **211** | **KNP_1020E1(F), KNP_1286D8(F)** |  |
| **233** | **80577759** | **bT95D6T7** | **AAAAATAGCCCAAGGATTTT** | **TCTTGGTCCTTAGATGGCTA** | **190** | **KNP_1286D8(F)** |  |
| **234** | **80640796** | **bT78I20T7** | **AGTTTAAATGGGTTGTGCTG** | **AAGAGCCTTCCTTTTTCAAT** | **199** | **KNP_768E9(F)** |  |
| **235** | **80690782** | **bE192L10T7** | **TGTTTTTAACACTTTGCTTTGT** | **TTTTGGAAGGTTTAAGCTACA** | **195** | **KNP_768E9(F), KNP_1048G11(U)** | **CH242_428L16(U)** |
| **236** | **80777364** | **bT78I20SP6** | **AAACTGCAGTGGATGTTAATC** | **AAATCACTCTGGGAGTTGTG** | **172** | **KNP_1048G11(U)** | **CH242_428L16(U)** |
| **237** | **80898750** | **bE315B17T7** | **TATGAGGAGACCTGGAAATG** | **GTTGTGGTTCCAGTTGCTAT** | **199** | **not screened** | **CH242_428L16(U), CH242_77M12(U)** |
| **238** | **80938959** | **bE140E23T7** | **GGCACAGAGAAGTTGGTACT** | **CTTCCAGTAATGGCGATAAT** | **200** | **not screened** | **CH242_77M12(U)** |
| **239** | **81031368** | **bT261M7SP6** | **CTGCTTGAACTACGGTTTTT** | **TGAAGACGGAATTGAACTTT** | **205** | **not screened** | **CH242_77M12(U), CH242_474E2(U)** |
| **240** | **81133895** | **bT146O17T7** | **CAGAATGTCAGGGCATAAAT** | **AATGTTCACCAGGACAAGAC** | **193** | **KNP_1645D7(F)** | **CH242_474E2(U)** |
| **241** | **81204969** | **bE79L17T7** | **TCCTGGTGATAGATGAGACC** | **ATTGGTTCTGTCAAAGGCTA** | **196** | **KNP_1645D7(F)** | **CH242_9I5(U)** |
| **242** | **81260991** | **bT117I4SP6** | **CAAAGACACATTCCTTGGTT** | **TTTGAAAAGGATTACCCAAA** | **197** | **not screened** | **CH242_9I5(U), CH242_394G14(U)** |
| **243** | **81295839** | **bT225I20T7** | **AACCATTAAGGGGAAGAGAG** | **CAGAAAACTGAATCCCTTTG** | **198** | **not screened** | **CH242_9I5(U), CH242_394G14(U)** |
| **244** | **81351748** | **bT119D18SP6** | **TTTATGGGTCTTTTCTCCAA** | **GGCCAACTCCATACAACTAC** | **200** | **KNP_1204A3(F)** | **CH242_394G14(U)** |
| **245** | **81432227** | **bE242K11T7** | **ACGTGCTTGTTAGAGGAAGA** | **AGTTCTGTGGAAACGAGAAA** | **204** | **KNP_1204A3(F), KNP_1405A7(U)** |  |
| **246** | **81501582** | **bE206P13SP6** | **CAGGAAATACAGGGATGAAA** | **TAAACAATGAATGGCACAAG** | **194** | **KNP_1405A7(U)** |  |
| **247** | **81583620** | **bT49O2T7** | **CCTGGAAGTCATTTTGTAGG** | **CACATCCAAAGAAAGGAGAC** | **193** | **KNP_181A3(F)** |  |
| **248** | **81643028** | **bE5B14SP6** | **TGGGTCAGTAAGAAGCCTAA** | **TTACCCAAAACAGATCATCC** | **202** | **not screened** | **CH242_375P3(U)** |
| **249** | **81712983** | **bT91H5T7** | **TGGCCAGTTACTTTTGTTTT** | **GACCACAAACCTCCTAACAA** | **197** | **KNP_1528B2(F)** | **CH242_375P3(U)** |
| **250** | **81773400** | **bT236G11SP6** | **CAAAATTGTACAGCAGACCA** | **TTGCAGGAACCGTATCTAAT** | **204** | **KNP_1528B2(F), KNP_701D10(U)** | **CH242_375P3(U)** |
| **251** | **81836496** | **bE141K5SP6** | **GCTAACCAAGTTCAGCAAGT** | **TTCTCTCCTCTCTCCTTTCC** | **161** | **KNP_701D10(U), KNP_743D6(U)** |  |
| **252** | **81908362** | **bE85K12SP6** | **TCGTCTAATTTGGTGAGATG** | **TGAGTCCCCCAAGTTCAT** | **150** | **KNP_743D6(U)** |  |
| **253** | **81977151** | **bE303H21T7** | **GGTATAGAGAAGCACCTGGA** | **TGACATCATTTTCTTTGTGC** | **200** | **KNP_743D6(U), KNP_602D5(F)** |  |
| **254** | **82037722** | **bE161P3SP6** | **TTTGCAAGTTGAGAGGAAAT** | **ACTTTTTCTTGCTGTGGGTA** | **194** | **KNP_602D5(F)** |  |
| **255** | **82101219** | **bT149E22T7** | **TCATGTTCTGACACCAGCTA** | **AGAAAGATGAGCAACCACTG** | **200** | **KNP_1563D9(U)** |  |
| **256** | **82159171** | **bE261I3SP6** | **GTCTTTGTCATGTGGTCCTT** | **CAGCAACCTCATTAATGTCA** | **200** | **KNP_1253B1(F)** |  |
| **257** | **82223480** | **bT208F7T7** | **GAAGGATGTGGAATGACACT** | **AACCCCTCTGAGAGAAAAAT** | **190** | **KNP_1253B1(F), KNP_76G10(F)** |  |
| **258** | **82262642** | **bE2K23SP6** | **GAAAGAAAGAGGTTGGTAAGC** | **ATAATTAATGCTGCCAGGAA** | **199** | **KNP_1253B1(F), KNP_76G10(F)** |  |
| **259** | **82295214** | **bT52K18SP6** | **ACCCTGATTCACAGAGTGAG** | **AAAAATCCTCTTGCTGTTTG** | **199** | **KNP_1253B1(F), KNP_76G10(F)** |  |
| **260** | **82351382** | **bE119G14T7** | **TTCATGAATTTGTTGCTTCA** | **CCATGGTTTTAAGCACTTTT** | **190** | **KNP_76G10(F), KNP_995H5(F)** |  |
| **261** | **82415605** | **bE267E13SP6** | **ACTTGACTACTCGGGAACAA** | **CTTTACCTGTCCAATGTGGT** | **201** | **KNP_995H5(F)** |  |
| **262** | **82479328** | **bT233G10SP6** | **AAGGCAAGCCATACTAACAG** | **CCACCTATTTCCTACAGCAG** | **202** | **KNP_995H5(F), KNP_1129D6(F)** |  |
| **263** | **82529177** | **bE26L17SP6** | **CTGCATCATTTCATTCACAG** | **GGATGGAGTTTTCAGAGATG** | **205** | **KNP_1129D6(F)** |  |
| **264** | **82579911** | **bE93F2SP6** | **TATGCACGCTATATGCACTC** | **TGAGACTTGCTTGGGATAAC** | **200** | **KNP_1129D6(F),KNP_1225B12(U)** |  |
| **265** | **82629486** | **bT132G21SP6** | **ACTTACCTGAGTCCAAAGCA** | **AGGGTTGTTGGAAAAGAGTT** | **194** | **KNP_1225B12(U)** |  |
| **266** | **82663847** | **bT77K15SP6** | **ACAAGTCCTCACCAGAAGAA** | **TTCTGTTTCCCACTCCTTTA** | **207** | **KNP_505G3(F), KNP_1563D9(F)** |  |
| **267** | **82708588** | **bE260I18SP6** | **ATGTGATTCAGTGAGCCTTT** | **TTATGATTTCTGCGGTTTTT** | **181** | **KNP_1445G8(F)** | **CH242_34G10(U)** |
| **268** | **82744396** | **bE288J4SP6** | **CAGTGAAAGAAATCCCAGAA** | **AAACTGTTGAATCACTTGGAA** | **192** | **KNP_1445G8(F)** | **CH242_34G10(U)** |
| **269** | **82820532** | **bT139B9SP6** | **ACCATGGCATAATAAACTGC** | **TTCCTTCATGACAGGGATAG** | **194** | **not screened** | **CH242_34G10(U)** |
| **270** | **82873566** | **bT123N3T7** | **AGAAACATTTTAAGCAGATCG** | **CCCGTTCAAACATAATGGTA** | **217** | **KNP_1352A10(U)** | **CH242_34G10(U), CH242_114O13(U)** |
| **271** | **82893163** | **bE99I12T7** | **TGTGAAATATGCTTGGATGA** | **TGCATGGCTTCTCTTAATTT** | **205** | **KNP_1352A10(U)** | **CH242_114O13(U)** |
| **272** | **82928697** | **bE12A2SP6** | **AAATCCAAGCCTTAACCTTC** | **ATCAGATCCAAAGCATTAGC** | **198** | **not screened** | **CH242_114O13(U)** |
| **273** | **83004889** | **bE114O13T7** | **AGCCACACAACAGTTTCTTT** | **CCATTGTCATGTGAAAGTCA** | **203** | **KNP_248G3(F)** | **CH242_114O13(U)** |
| **274** | **83034730** | **bE244L18T7** | **GCAGAAGGAGTGTTGCTATC** | **GCTATCTGCCTTTCTTACCA** | **182** | **KNP_248G3(F)** |  |
| **275** | **83095631** | **CH242_0012A2.f** | **CCAGAATGGCTATTTCTTTG** | **TACGTAGAAGACCCTGGAAA** | **192** | **KNP_248G3(F), KNP_570E6(F)** |  |
| **276** | **83152479** | **bE247C14SP6** | **TCAGCACCCTTCTCTCTTAG** | **CCCGTTCAAACATAATGGTA** | **198** | **not screened** | **CH242_183K20(U)** |
| **277** | **83216867** | **bE251B2T7** | **ATTGTTGGGACTTCTCTGTG** | **TAGGCATTTTCAGACATCCT** | **204** | **KNP_1685E3(F)** | **CH242_183K20(U)** |
| **278** | **83281381** | **bT80M23SP6** | **ATGCATCTCATCCATAGCTT** | **TAAACAATGGGTCTTTGTCC** | **191** | **KNP_1685E3(F), KNP_287G10(F)** |  |
| **279** | **83342956** | **bT131H1T7** | **TCCAGCTTCAGAGAAGAAAG** | **AATACTGGTGCTGGGATTTA** | **201** | **KNP_287G10(F)** | **CH242_150C4(U)** |
| **280** | **83580364** | **bE225F2SP6** | **AATACTTGCTGGCTTCTGTC** | **AATTTTAAAAGACAAAATGATGC** | **200** | **not screened** | **CH242_246N13(U)** |
| **281** | **83754393** | **bE186H9T7** | **TCCTGACTCTGGCTTTTAGA** | **AGAGAAACAAGGGTGCATTA** | **199** | **KNP_88C1(F)** | **CH242_292I3(U)** |
| **282** | **83797620** | **bE281I5SP6** | **CTCTTTTGACAGGGACTCAG** | **TTTAACCACAATTCCAAACC** | **196** | **KNP_88C1(F)** |  |
| **283** | **83856551** | **bE38N14SP6** | **TCAAGAATCCTGCTGTCTCT** | **GAGGGATAGATTTGGCTTTT** | **202** | **KNP_1443A1(F), KNP_701D10** | **CH242_326G7(U)** |
| **284** | **83915171** | **bT122M13T7** | **GAAAATATCCCCCTGTAACC** | **ACTTACATCGCAACACAACA** | **196** | **KNP_1443A1(F), KNP_701D10** | **CH242_326G7(U)** |
| **285** | **83972346** | **bE122F12T7** | **TCATCACTTTAAAATGTCCAAA** | **ATCATTCATTTGTTCCCAAG** | **170** | **KNP_1443A1(F), KNP_298B2(F)** | **CH242_326G7(U)** |
| **286** | **84033032** | **bE203N3T7** | **CTCAGAGGAGCAGAACAGAT** | **TACAGGAAGAAGGCAAAGAG** | **200** | **KNP_298B2(F), KNP_1814F9(U)** |  |
| **287** | **84090126** | **RPCI44_0383G23.f** | **ATATGTTCATAGCGCATTCC** | **TTTTCCATTATGCTCTAGCC** | **205** | **KNP_1814F9(U)** |  |
| **288** | **84123640** | **RPCI44_0416M23.f** | **TTTGATATTTTGGAGCCATT** | **GAAGCCAAACTGCTCATATT** | **199** | **KNP_1814F9(U)** | **CH242_121F17(U)** |
| **289** | **84155328** | **bT241M17SP6** | **ACCTTGTGGACTATGCAGAC** | **CATGTCTGGGGATATAAGGA** | **198** | **not screened** | **CH242_121F17(U)** |
| **290** | **84196296** | **bE131P11SP6** | **CATCACACACTCAGACCTTG** | **AGCAGCTCAGCTCAAGATAG** | **199** | **not screened** | **CH242_121F17(U)** |
| **291** | **84273607** | **bE262I16T7** | **AGGGGAACAAAATATGGAAT** | **ACTGGCTTGCTAATTCTTTG** | **201** | **KNP_1819D10(U)** |  |
| **292** | **84317067** | **bT89L6SP6** | **AAAAACCATGCTGATTGACT** | **TTGCCTTTTCTTGAGACATT** | **200** | **KNP_1819D10(U)** |  |
| **293** | **84349598** | **RPCI44_382N10.f** | **CCGTTTCCTATTTGAGATTG** | **GCCTCTATGAAAGAAAAGCA** | **250** | **KNP_1819D10(U)** |  |
| **294** | **84379894** | **bT93K10T7** | **GTTCCTAGTCGGATTCGTTA** | **CTCATATTTCAGGCCCATTA** | **199** | **KNP_1819D10(U), KNP_1722G11(U)** |  |
| **295** | **84408141** | **RPCI44_0432P12.f** | **GAATTCCAAAGACATTCAGG** | **GTTTAAAGGGATGCTTGCTA** | **187** | **KNP_1819D10(U), KNP_1722G11(U)** |  |
| **296** | **84450107** | **bT126J20T7** | **TTAAGCAACCAAAGACCATT** | **TCTCATAAGCCATTGTGTCA** | **200** | **KNP_1722G11(U)** |  |
| **297** | **84484062** | **bT226P4T7** | **TTTTCTGGGTGTCAGTAACC** | **TTTTAACAGCTAAGCCCATC** | **206** | **KNP_1722G11(U), KNP_1202D4(U)** |  |
| **298** | **84524040** | **bE283A10SP6** | **TTCACAGCAGATTTTCCTCT** | **GCTCAAGAAAAGCCAAAATA** | **200** | **KNP_1202D4(U)** | **CH242_72M21(U)** |
| **299** | **84584859** | **RPCI44_0401J2.f** | **GCCAGAGGGATACAGACATA** | **CTAGTATGCCATGCTGACTG** | **209** | **KNP_1736G11(U)** | **CH242_72M21(U)** |
| **300** | **84608914** | **bE91D13T7** | **ATTTCCTGCTCTTTTCAACA** | **ATTTCTACCCTGTGATGAGC** | **195** | **KNP_1736G11(U)** | **CH242_72M21(U)** |
| **301** | **84637229** | **RPCI44_0372N7.f** | **CAGAGGTGTGGTAGAGCTTC** | **ATCAAGCCCTCTTTCTCTTT** | **169** | **KNP_1736G11(U)** | **CH242_72M21(U)** |
| **302** | **84665723** | **bE178H3T7** | **TCCCAGAGACAGAGTAGCAT** | **AACCATCCCTGCAGTTAATA** | **200** | **KNP_1736G11(U)** | **CH242_72M21(U)** |
| **303** | **84702384** | **bE91M24SP6** | **ACAAGATCCTGCGAAGACTA** | **GCCATACTTGAAAGGAATTG** | **204** | **KNP_240H2(U)** |  |
| **304** | **84733210** | **RPCI44_0396K19.f** | **TCAGAAAGACACACAGGTCA** | **TTGGCAGTGCTACAGTTATG** | **184** | **KNP_240H2(U)** |  |
| **305** | **84770132** | **RPCI44_0327N14.f** | **TCTCTGGAAAGCACTGGTAT** | **TCCATGGATCTTTTGTTCAT** | **198** | **KNP_240H2(U), KNP_1294G4(U)** |  |
| **306** | **84803948** | **bE136D13SP6** | **ACCTTTGAGCTATGCAGTGT** | **ACTGTTCTGGTTCCACACAT** | **196** | **KNP_240H2(U), KNP_1294G4(U)** | **CH242_520E12(U)** |
| **307** | **84886282** | **bE252P3SP6** | **AACATCAGTGGCAAAGAAAC** | **CCAGACCCAAAGTGTTTTAT** | **208** | **KNP_1294G4(U)** | **CH242_520E12(U)** |
| **308** | **84913331** | **bE129J6T7** | **CATGATGGGAACTCCATAGT** | **TTTCATTATTGGAAGGTTGC** | **196** | **not screened** | **CH242_520E12(U)** |
| **309** | **84956579** | **bT174E23T7** | **TGGGCAGGCTCTAAGTAATA** | **CAGCTCCTTAACCAACTGAG** | **208** | **not screened** | **CH242_520E12(U)** |
| **310** | **84994771** | **bE264C8T7** | **GAGAGTGCTGGGTTAATTTG** | **AAGGAACCAGTGATGCTATG** | **203** | **KNP_461A2(U)** | **CH242_520E12(U)** |
| **311** | **85032963** | **bE271C7SP6** | **CCTTCAAATGAGAACTCAGC** | **GTCCCTAAACACCTGCATTA** | **200** | **KNP_461A2(U)** | **CH242_520E12(U), CH242_431N22(U)** |
| **312** | **85068593** | **bE49M13T7** | **CTGTGTAGAAACGTGGAATG** | **CAGGAGGCAAATAAGTTCTG** | **181** | **KNP_461A2(U)** | **CH242_431N22(U)** |
| **313** | **85118765** | **bE269L23T7** | **GACATTGGAGCAAAGAAGTC** | **AGTTCCACACAGCAAATTCT** | **194** | **KNP_461A2(U)** | **CH242_431N22(U)** |
| **314** | **85170815** | **bT76B21T7** | **AGAACAGAGCCTTCAGATCA** | **AACCCCATGATGTAAAGATG** | **200** | **KNP_1511G3(F)** | **CH242_431N22(U)** |
| **315** | **85233640** | **bE138F23SP6** | **AGATCTGGTGTGGTTGTAGC** | **CAGTTTGTATGTGCAGGAGA** | **206** | **KNP_1511G3(F)** |  |

^*^ (F): completely finished BAC clone

^**^ (U): unfinished BAC clone
