# Supplementary material for: Neuronal Genes for Subcutaneous Fat Thickness in Human and Pig Are Identified by Local Genomic Sequencing and Combined SNP Association Study
Source: PLoS One. 2011 Feb 2;6(2):e16356. doi: 10.1371/journal.pone.0016356 (PMC3032728; doi:10.1371/journal.pone.0016356)
Supplement: Table S3 — List of gene annotation in the pig 18.2 Mb region. (DOC) [file pone.0016356.s003.doc]

**Table S3.** List of gene annotation in the pig 18.2 Mb region

| Gene symbol | Direction | Start | End | Bio Type |
| --- | --- | --- | --- | --- |
| CYP4A11 | + | 3421 | 14455 | protein coding |
| MCOLN2 | + | 67947 | 127327 | protein coding |
| LPAR3 | + | 175647 | 228892 | protein coding |
| SSX2IP | + | 366130 | 395785 | protein coding |
| CTBS | + | 457578 | 491987 | protein coding |
| SPATA1 | - | 490429 | 534934 | protein coding |
| GNG5 | + | 534904 | 542721 | protein coding |
| BXDC5 | - | 543798 | 558492 | protein coding |
| DNASE2B | - | 609824 | 624020 | protein coding |
| UOX | + | 624209 | 655692 | protein coding |
| SAMD13 | - | 682187 | 723213 | protein coding |
| PRKACB | - | 805917 | 924370 | protein coding |
| ENSSSCG00000003759 | - | 956952 | 957074 | protein coding* |
| TTLL7 | + | 997022 | 1148089 | protein coding |
| U6 (ENSSSCT00000020739) | + | 1436113 | 1436219 | snRNA |
| LPHN2 | - | 3011105 | 3656509 | protein coding |
| U1 | + | 4196001 | 4196159 | snRNA |
| ELTD1 | + | 5567864 | 5668010 | protein coding |
| IFI44 | - | 5839333 | 5855968 | protein coding |
| IFI44L | - | 5865999 | 5885250 | protein coding |
| PTGFR | - | 5937387 | 5982453 | protein coding |
| pseudogene(SURF6) | + | 6220943 | 6221819 | pseudogene |
| GIPC2 | - | 6299636 | 6381458 | protein coding |
| U6 (ENSSSCG00000018943) | + | 6346544 | 6346650 | snRNA |
| DNAJB4 | - | 6406780 | 6419005 | protein coding |
| U6 (ENSSSCG00000018423) | + | 6430734 | 6430833 | snRNA |
| FUBP1 | + | 6444811 | 6478210 | protein coding |
| NEXN | - | 6483929 | 6541255 | protein coding |
| FAM73A | - | 6552881 | 6637204 | protein coding |
| USP33 | + | 6649590 | 6714241 | protein coding |
| ZZZ3 | + | 6731776 | 6866564 | protein coding |
| U6 (ENSSSCG00000019419) | + | 6831393 | 6831499 | snRNA |
| AK5 | - | 6872099 | 7144596 | protein coding |
| PIGK | + | 7182537 | 7294974 | protein coding |
| ST6GALNAC5 | - | 7324954 | 7508413 | protein coding |
| ST6GALNAC3 | - | 7740568 | 8287459 | protein coding |
| ASB17 | + | 8469023 | 8486637 | protein coding |
| MSH4 | - | 8496436 | 8594800 | protein coding |
| RABGGTB | - | 8596347 | 8604020 | protein coding |
| SNORD45 (ENSSSCG00000019699) | - | 8602966 | 8603036 | snoRNA |
| SNORD45 (ENSSSCG00000019290) | - | 8603547 | 8603629 | snoRNA |
| SNORD45 (ENSSSCG00000019181) | - | 8604196 | 8604274 | snoRNA |
| ACADM | - | 8612667 | 8646884 | protein coding |
| SLC44A5 | + | 8666626 | 9067600 | protein coding |
| LHX8 | - | 9132625 | 9159760 | protein coding |
| TYW3 | - | 9485905 | 9502512 | protein coding |
| CRYZ | + | 9502610 | 9528282 | protein coding |
| C1orf173 | + | 9571276 | 9664930 | protein coding |
| TNNI3K | - | 9702605 | 9972834 | protein coding |
| LRRC53 | + | 9768665 | 9781367 | protein coding |
| B9TRW8 | - | 10000580 | 10009070 | protein coding* |
| FPGT | - | 10005575 | 10009070 | protein coding |
| LRRIQ3 | + | 10009161 | 10200753 | protein coding |
| pseudogene (YARS2) | - | 11060485 | 11060970 | pseudogene |
| NEGR1 | + | 11783027 | 12694533 | protein coding |
| ENSSSCG00000003785 | - | 12236820 | 12237843 | protein coding* |
| ZRANB2 | + | 12976220 | 12996957 | protein coding |
| mir186 | + | 12992529 | 12992614 | miRNA |
| PTGER3 | + | 13008388 | 13091328 | protein coding |
| CTH | - | 13504380 | 13541728 | protein coding |
| ANKRD13C | + | 13571667 | 13672377 | protein coding |
| SFRS11 | - | 13677356 | 13725123 | protein coding |
| LRRC40 | + | 13725174 | 13771796 | protein coding |
| LRRC7 | - | 13794158 | 14330161 | protein coding |
| DEPDC1 | + | 15192641 | 15217648 | protein coding |
| RPE65 | + | 15241318 | 15263197 | protein coding |
| GPR177 | + | 15458161 | 15565107 | protein coding |
| DIRAS3 | + | 15611828 | 15614308 | protein coding |
| ENSSSCG00000003796 | - | 15617776 | 15618260 | protein coding* |
| GNG12 | + | 15872244 | 15949572 | protein coding |
| U6 | - | 15931468 | 15931572 | snRNA |
| GADD45A | - | 15974550 | 15977875 | protein coding |
| SERBP1 | + | 16176161 | 16191766 | protein coding |
| IL12RB2 | - | 16203410 | 16269120 | protein coding |
| pseudogene (ENSSSCG00000003800) | - | 16304144 | 16304963 | pseudogene |
| IL23R | - | 16327318 | 16390513 | protein coding |
| C1orf141 | + | 16441748 | 16496073 | protein coding |
| SLC35D1 | + | 16523049 | 16579304 | protein coding |
| MIER1 | - | 16597509 | 16667941 | protein coding |
| WDR78 | + | 16668011 | 16741552 | protein coding |
| INSL5 | + | 16755930 | 16756436 | protein coding |
| TCTEX1D1 | - | 16773611 | 16800455 | protein coding |
| SGIP1 | - | 16808759 | 17038051 | protein coding |
| PDE4B | - | 17154331 | 17706827 | protein coding |
| LEPR | - | 17784071 | 17880559 | protein coding |
| LEPROT | - | 17960622 | 17973280 | protein coding |
| DNAJC6 | - | 17976243 | 18140923 | protein coding |
| AK3L1 | - | 18167591 | 18243658 | protein coding |

*novel genes
