# Supplementary material for: Neuronal Genes for Subcutaneous Fat Thickness in Human and Pig Are Identified by Local Genomic Sequencing and Combined SNP Association Study
Source: PLoS One. 2011 Feb 2;6(2):e16356. doi: 10.1371/journal.pone.0016356 (PMC3032728; doi:10.1371/journal.pone.0016356)
Supplement: Table S4 — List of SNPs significantly associated with the backfat thickness trait in the 18.2 Mb region. (DOC) [file pone.0016356.s004.doc]

**Table S4. List of SNPs significantly associated with the backfat thickness trait in the 18.2 Mb region.**

| **GENE** | **Nearby SNPs** | **DIS (kb)** | ***p-*value** |
| --- | --- | --- | --- |
| NEGR1 | ALGA0122230 | 591.1 | 3.99E-07 |
| SLC44A5 | ASGA0029572 | Intron | 1.48E-06 |
|  | ASGA0029573 | Intron | 6.42E-05 |
| PDE4B | MARC0083918 | Intron | 1.68E-06 |
| LPHN2 | ALGA0036729 | 268.2 | 2.59E-06 |
|  | H3GA0018809 | 346.6 | 3.17E-06 |
|  | ALGA0036740 | Intron | 4.29E-06 |
|  | H3GA0018823 | 267.4 | 4.38E-06 |
|  | H3GA0018816 | 199.9 | 5.14E-06 |
|  | ALGA0036776 | 357.3 | 6.14E-06 |
|  | ASGA0091848 | 109.6 | 6.70E-06 |
|  | ASGA0029430 | 6.2 | 8.84E-06 |
|  | H3GA0018820 | 37.4 | 9.38E-06 |
|  | ASGA0029422 | Intron | 1.04E-05 |
|  | ALGA0036742 | Intron | 1.38E-05 |
|  | MARC0035827 | Intron | 1.39E-05 |
|  | ALGA0036763 | 244.4 | 1.45E-05 |
|  | DRGA0006798 | 217.1 | 2.02E-05 |
|  | ALGA0116715 | 11.3 | 4.49E-05 |
|  | ASGA0029438 | 309.8 | 5.01E-05 |
|  | SIRI0001502 | 626.5 | 5.65E-05 |
|  | INRA0022280 | 323.8 | 5.79E-05 |
|  | ASGA0098556 | 34.7 | 5.90E-05 |
|  | ALGA0119841 | 856 | 7.59E-05 |
|  | ALGA0106465 | 45.2 | 8.93E-05 |
|  | ALGA0036715 | 552.5 | 1.03E-04 |
|  | ASGA0029427 | 175.3 | 1.20E-04 |
|  | MARC0063627 | 67.9 | 1.37E-04 |
| ELTD1 | MARC0045573 | 481.8 | 4.20E-06 |
|  | ALGA0121932 | 299 | 4.26E-06 |
|  | DRGA0006809 | 506.1 | 4.38E-06 |
|  | ALGA0036794 | 589.4 | 5.21E-06 |
|  | MARC0029787 | 538 | 5.61E-06 |
|  | DRGA0006812 | Intron | 1.07E-05 |
|  | MARC0059867 | 454.8 | 1.20E-05 |
|  | INRA0022295 | 526.2 | 1.37E-05 |
|  | ALGA0114621 | 247.6 | 1.30E-04 |
| ST6GALNAC3 | MARC0083766 | Intron | 4.69E-06 |
|  | MARC0046979 | Intron | 7.76E-06 |
| ST6GALNAC5 | ALGA0104531 | 36.5 | 1.51E-05 |
|  | ALGA0036880 | Intron | 5.68E-05 |
| FAM73A | ASGA0029495 | Intron | 3.10E-05 |
| TTLL7 | DRGA0006767 | 679 | 6.56E-05 |
|  | H3GA0018787 | 243.3 | 7.13E-05 |
|  | ALGA0036710 | 899.9 | 7.16E-05 |
|  | ALGA0036705 | 721.3 | 7.53E-05 |
|  | ASGA0029405 | 725.7 | 8.54E-05 |
| C1orf141 | ASGA0029677 | Intron | 1.12E-04 |
| PTGFR | MARC0101649 | 60.6 | 1.26E-04 |
| ZZZ3 | H3GA0018851 | Intron | 1.32E-04 |
| GIPC2 | ASGA0029488 | Intron | 1.61E-04 |
|  | MARC0042529 | Intron | 2.43E-04 |
