# Supplementary material for: Neuronal Genes for Subcutaneous Fat Thickness in Human and Pig Are Identified by Local Genomic Sequencing and Combined SNP Association Study
Source: PLoS One. 2011 Feb 2;6(2):e16356. doi: 10.1371/journal.pone.0016356 (PMC3032728; doi:10.1371/journal.pone.0016356)
Supplement: Table S5 — List of SNPs associated with subscapular skin-fold thickness at the threshold of genomic control-corrected p-value 0.01. (DOC) [file pone.0016356.s005.doc]

**Table S5.** List of SNPs associated with subscapular skin-fold thickness at the threshold of genomic control-corrected *p*-value 0.01

| Gene | SNP | Distance(Kb) | Allele* | MAF | Raw *p*-value | GC-corrected  *p*-value | FDR  *q* value |
| --- | --- | --- | --- | --- | --- | --- | --- |
| FAM73A | rs4121165 | Intron | T/C | 4.49E-01 | 1.61E-05 | 1.75E-05 | 3.76E-02 |
| AK5 | rs1874817 | Intron | T/C | 4.36E-01 | 5.33E-04 | 5.64E-04 | 6.04E-01 |
|  | rs2803152 | Intron | T/C | 4.38E-01 | 9.87E-04 | 1.04E-03 | 6.77E-01 |
|  | rs12093263 | Intron | A/G | 4.37E-01 | 1.22E-03 | 1.28E-03 | 6.77E-01 |
|  | rs6704141 | Intron | T/C | 4.18E-01 | 3.77E-03 | 3.92E-03 | 6.77E-01 |
| ELTD1 | rs12142366 | Intron | G/T | 2.10E-01 | 2.16E-03 | 2.26E-03 | 6.77E-01 |
|  | rs7552978 | Intron | T/C | 2.09E-01 | 2.27E-03 | 2.37E-03 | 6.77E-01 |
|  | rs7414397 | 35.7 | T/A | 3.95E-01 | 6.24E-03 | 6.47E-03 | 6.77E-01 |
|  | rs6665405 | 34.7 | A/G | 3.95E-01 | 6.34E-03 | 6.57E-03 | 6.77E-01 |
| SGIP1 | rs699253 | 58.7 | A/G | 2.37E-01 | 4.03E-03 | 4.19E-03 | 6.77E-01 |
| ACADM | rs7552289 | 9.1 | C/G | 6.35E-02 | 4.24E-03 | 4.41E-03 | 6.77E-01 |
|  | rs1463812 | 8.1 | T/C | 7.19E-02 | 9.48E-03 | 9.80E-03 | 6.77E-01 |
| RABGGTB | rs1146610 | 10.6 | A/G | 7.15E-02 | 5.93E-03 | 6.15E-03 | 6.77E-01 |
| SLC44A5 | rs211719 | 29.0 | G/A | 7.86E-02 | 8.26E-03 | 8.55E-03 | 6.77E-01 |
|  | rs396070 | 44.7 | T/A | 7.83E-02 | 8.90E-03 | 9.20E-03 | 6.77E-01 |
|  | rs211713 | 31.4 | C/T | 7.85E-02 | 8.91E-03 | 9.21E-03 | 6.77E-01 |
|  | rs399160 | 37.9 | A/G | 7.84E-02 | 9.10E-03 | 9.40E-03 | 6.77E-01 |
| GIPC2 | rs483760 | Intron | A/G | 4.78E-01 | 5.94E-03 | 6.17E-03 | 6.77E-01 |
| TTLL7 | rs6576947 | 71.0 | T/A | 3.14E-01 | 6.23E-03 | 6.46E-03 | 6.77E-01 |
|  | rs6576946 | 71.4 | T/C | 3.14E-01 | 6.52E-03 | 6.76E-03 | 6.77E-01 |
| NEGR1 | rs2630425 | Intron | T/G | 2.35E-01 | 6.31E-03 | 6.54E-03 | 6.77E-01 |
|  | rs1486090 | Intron | G/A | 2.34E-01 | 8.38E-03 | 8.67E-03 | 6.77E-01 |
| ST6GALNAC3 | rs1013629 | 26.6 | G/A | 8.67E-02 | 6.36E-03 | 6.60E-03 | 6.77E-01 |
|  | rs1486932 | Intron | T/A | 1.08E-01 | 7.27E-03 | 7.53E-03 | 6.77E-01 |
| LRRC7 | rs10789293 | 335.0 | T/C | 7.82E-02 | 8.00E-03 | 8.28E-03 | 6.77E-01 |
| IL23R | rs17375018 | Intron | A/G | 3.20E-01 | 8.49E-03 | 8.78E-03 | 6.77E-01 |
|  | rs6664119 | Intron | C/T | 3.61E-01 | 9.18E-03 | 9.49E-03 | 6.77E-01 |
| GNG12 | rs12083080 | 14.0 | C/T | 2.17E-01 | 8.79E-03 | 9.09E-03 | 6.77E-01 |
|  | rs2246675 | Intron | C/T | 2.18E-01 | 8.99E-03 | 9.29E-03 | 6.77E-01 |
|  | rs11579057 | 24.9 | A/G | 2.17E-01 | 9.12E-03 | 9.43E-03 | 6.77E-01 |
|  | rs12563734 | 2.0 | A/C | 2.17E-01 | 9.15E-03 | 9.46E-03 | 6.77E-01 |

*The alleles are shown as major/minor allele.
