# Supplementary material for: Neuronal Genes for Subcutaneous Fat Thickness in Human and Pig Are Identified by Local Genomic Sequencing and Combined SNP Association Study
Source: PLoS One. 2011 Feb 2;6(2):e16356. doi: 10.1371/journal.pone.0016356 (PMC3032728; doi:10.1371/journal.pone.0016356)
Supplement: Table S6 — List of SNPs associated with suprailiac skin-fold thickness at the threshold of genomic control-corrected p-value 0.01. (DOC) [file pone.0016356.s006.doc]

**Table S6. List of SNPs associated with suprailiac skin-fold thickness at the threshold of genomic control-corrected *p*-value 0.01**

| Gene | SNP | Distance (Kb) | Allele* | MAF | Raw *p*-value | GC-corrected  *p*-value | FDR  *q* value |
| --- | --- | --- | --- | --- | --- | --- | --- |
| FAM73A | rs4121165 | Intron | T/C | 4.49E-01 | 4.55E-05 | 3.10E-04 | 4.41E-01 |
| LPHN2 | rs12140830 | 626.1 | A/G | 2.11E-01 | 6.50E-05 | 4.12E-04 | 4.41E-01 |
| AK5 | rs6704141 | Intron | T/C | 4.18E-01 | 6.64E-04 | 2.61E-03 | 8.06E-01 |
|  | rs1874817 | Intron | T/C | 4.36E-01 | 2.00E-03 | 6.29E-03 | 8.06E-01 |
| TTLL7 | rs10782820 | 77.1 | C/G | 1.74E-01 | 6.73E-04 | 2.64E-03 | 8.06E-01 |
|  | rs10489506 | 66.5 | G/C | 1.79E-01 | 1.30E-03 | 4.45E-03 | 8.06E-01 |
|  | rs1040576 | 60.8 | C/A | 1.79E-01 | 1.90E-03 | 6.01E-03 | 8.06E-01 |
|  | rs12057556 | 70.8 | A/T | 1.77E-01 | 1.91E-03 | 6.06E-03 | 8.06E-01 |
|  | rs17129475 | 80.1 | G/C | 1.65E-01 | 2.36E-03 | 7.17E-03 | 8.06E-01 |
|  | rs11163848 | 55.6 | A/G | 1.66E-01 | 2.93E-03 | 8.50E-03 | 8.41E-01 |
|  | rs12082518 | 42.3 | T/C | 1.66E-01 | 3.27E-03 | 9.29E-03 | 8.41E-01 |
|  | rs7540335 | 120.6 | A/G | 9.11E-02 | 3.36E-03 | 9.51E-03 | 8.41E-01 |
| LRRC7 | rs17131208 | Intron | T/C | 1.38E-01 | 8.30E-04 | 3.11E-03 | 8.06E-01 |
|  | rs17131218 | Intron | G/A | 1.39E-01 | 1.03E-03 | 3.71E-03 | 8.06E-01 |
|  | rs17131204 | Intron | A/G | 1.39E-01 | 1.17E-03 | 4.10E-03 | 8.06E-01 |
|  | rs17131215 | Intron | T/C | 1.39E-01 | 1.18E-03 | 4.12E-03 | 8.06E-01 |
| GADD45A | rs12083789 | 106.0 | A/T | 4.36E-01 | 8.54E-04 | 3.19E-03 | 8.06E-01 |
| NEGR1 | rs2630388 | Intron | G/A | 2.80E-01 | 1.12E-03 | 3.95E-03 | 8.06E-01 |
|  | rs1486090 | Intron | G/A | 2.34E-01 | 1.53E-03 | 5.07E-03 | 8.06E-01 |
|  | rs2630425 | Intron | T/G | 2.35E-01 | 1.90E-03 | 6.02E-03 | 8.06E-01 |
|  | rs1954600 | Intron | T/C | 2.37E-01 | 2.09E-03 | 6.49E-03 | 8.06E-01 |
|  | rs2821282 | Intron | T/G | 2.83E-01 | 2.51E-03 | 7.52E-03 | 8.06E-01 |
| DNAJC6 | rs7541003 | Intron | T/A | 4.14E-01 | 2.25E-03 | 6.90E-03 | 8.06E-01 |
| SAMD13 | rs7528615 | 31.1 | A/G | 3.22E-01 | 3.12E-03 | 8.95E-03 | 8.41E-01 |

*The alleles are shown as major/minor allele.
